# Identification and genetic diversity analysis of high-yielding charcoal rot resistant soybean genotypes

**DOI:** 10.1038/s41598-023-35688-2

**Published:** 2023-06-01

**Authors:** Pawan K. Amrate, M. K. Shrivastava, M. S. Bhale, Nisha Agrawal, Giriraj Kumawat, M. Shivakumar, Vennampally Nataraj

**Affiliations:** 1 Department of Plant Breeding and Genetics, Jawaharlal Nehru Krishi VishwaVidyalaya, Jabalpur, Madhya Pradesh 482004 India; 2 ICAR-Indian Institute of Soybean Research, Indore, Madhya Pradesh 452001 India

**Keywords:** Molecular biology, Plant sciences

## Abstract

Charcoal rot disease caused by *Macrophomina phaseolina* (Tassi) Goid is one of the most devastating diseases in soybean in India. During 2018, 226 diverse soybean genotypes were evaluated for genetic resistance under hot-spot conditions. Out of them, a subset of 151 genotypes were selected based on Percent Disease Incidence (PDI) and better agronomic performance. Out of these 151 genotypes evaluated during 2019, 43 genotypes were selected based on PDI and superior agronomic performance for further field evaluation and molecular characterization. During 2020 and 2021, these forty-three genotypes, were evaluated for PDI, Area Under Disease Progress Curve (AUDPC), and grain yield. In 2020, genotype JS 20-20 showed least PDI (0.42) and AUDPC (9.37).Highest grain yield was recorded by the genotype JS 21-05 (515.00 g). In 2021, genotype JS 20-20 exhibited least PDI (0.00) and AUDPC (0.00).Highest grain yield was recorded in JS 20-98 (631.66 g). Across both years, JS 20-20 had the least PDI (0.21) and AUDPC (4.68), while grain yield was highest in JS 20-98 (571.67 g). Through MGIDI (multi-trait genotype-ideotype distance) analysis, JS 21-05 (G19), JS 22-01 (G43), JS 20-98 (G28) and JS 20-20 (G21) were identified as the ideotypes with respect to the traits that were evaluated. Two unique alleles, Satt588 (100 bp) on linkage group K (Chromosome no 9) and Sat_218 (200 bp) on linkage group H (Chromosome no 12), were specific for thetwo resistant genotypes JS 21-71and DS 1318, respectively. Through cluster analysis, it was observed that the genotypes bred at Jabalpur were more genetically related.

## Introduction

Soybean (*Glycine max* L. Merril) is the foremost important leguminous crop in the world, contributing 25% edible oil and two-thirds of the protein in livestock feed^1^. India ranks fifth in the world’s edible oil market; nevertheless, 50% of its edible oil demand is met through imports^1^. Soybean is the mainIndian oilseed crop with a production of 10.45 million metric tons in an area of 12.7 million hectares^2^. With an export share of USD 82,10,524, India has been a key player of soybean defatted oil cake (DOC) in international markets^3^. Soybean production in India is under rainfed conditions which results in various forms of biotic stresses such as drought. Therefore, soybean productivity has been much lower compared to the major growing countries like U.S.A and Brazil^1^.

Charcoal rot disease caused by *Macrophomina phaseolina* (Tassi) Goid is the most devastating soybean disease in India, causing as much as a 77% yield loss^4^. An estimate of 39.2 thousand metric tones’ yield loss is attributed to this disease in India^5^. Although drought/drought-like conditions aggravate this disease, significant yield losses are reported even under irrigated conditions^6,7^.Soybean is vulnerable to this disease across all growth stages, but disease aggravation is often observed during reproductive stages^6^. Greyish black appearance of the lower stem and root tissue and presence of numerous black microsclerotia are major diagnostic symptoms of soybean charcoal rot disease^8,9^.

*M. phaseolina* is a necrotrophic fungal pathogen having a broad host range including economical crop species like soybean (*Glycine max,* L.), sorghum (*Sorghum bicolor* (L.) Moench), maize (*Zea mays,* L.), cotton (*Gossypium hirsutum* L.) etc.^10,11^. Genetic resistance is the most effective and eco-friendly means of managing this disease^12^.However, breeding and genomics of charcoal rot resistance has been limited due to the quantitative nature of host-plant resistance, the pathogenic variability and multi-dimensional mode of its pathogenicity^13^.

Though artificial screening is less tedious, field screening should still be considered, since it is the ultimate environment where the crop is grown^14^. Area Under Disease Progress curve has been extensively employed in assessment of partial or quantitative disease resistance under field conditions, for temporal integration of disease progress in relation to the growth and development of host plants^15^. Percent Disease Index (PDI) and AUDPC have often been used for charcoal rot disease evaluation^16,17^.

Indian soybean mega-varieties such as JS 95-60 and JS 93-05 and many other varieties are highly susceptible to charcoal rot disease. Despite the importance of this disease in India, only a few reports on charcoal rot resistance are available^16,18^. Simultaneous selection for grain yield and resistance/tolerance to different forms of stresses helps in development of superior varieties. Studies on the effect of charcoal rot disease incidence on grain yield revealed that the association between disease incidence and grain yield was not always negative and the trends could vary across genotypes and environments^19^. Further, some soybean genotypes, which are sensitive to charcoal rot disease showed no yield penalty^19^. Similarly, in case of sorghum, no significant positive correlation between charcoal rot disease incidence and yield has been observed^20,21^. However, grain yield under high-disease pressure can be vital in soybean breeding for resistance under charcoal rot stress.

Precise estimation of genotypic values is critical for selection and recommendation of any genotype. A mixed-model based best linear unbiased prediction (BLUP) method estimates the random effects^22^. The shrinkage property of BLUP model tends to narrow the difference between actual and predicted genotypic values^23^. Superiority of BLUP method for prediction accuracy, over Best Linear Unbiased Estimation (BLUE) and other models has been demonstrated through several studies^11,24,25^.

Breeders often target multiple traits during the selection process, in addition to the yield performance. Multi-trait-based selections are often carried out using a linear-selection index: Smith-Hazel (SH) index^26^. However, the multi-collinearity effect and irrational allocation of weightage coefficients to the traits under study, renders the SH index ineffective in achieving the desired genetic gain^27^. A recent multivariate and BLUP based selection index, MGIDI that negates these drawbacks was found to outperform the SH index^28^.

The genetic base of the soybean genotypes/cultivars bred in India is very narrow. An insight into their genetic base through molecular diversity analysis will be more reliable and stable as compared to the conventional, phenotype-based diversity analysis. Genetically diverse genotypes identified through molecular markers have been employed as parents in breeding programs^29,30^. Simple sequence repeat (SSR) markers have been extensively employed in genetic diversity assessment studies in soybean^31–33^. This current investigation was for identification and molecular characterization of high-yielding charcoal rot resistant soybean genotypes.

## Materials and methods

### Preliminary screening for charcoal rot resistance

During 2018, a total of 226 soybean genotypes including varieties, breeding lines and exotic accessions were evaluated for charcoal rot resistance under hot spot conditions at J.N.K.V.V, Jabalpur, India. The experimental design used was an augmented block design containing seven blocks. Out of 226 genotypes, subsets of 151 genotypes were selected based on disease reaction and better agronomic performance. This subsets was evaluated in 2019 using an augmented block design containing six blocks.

During both the years, genotypes were sown in two rows three meters long. Four checks-JS 20-29, JS 335, JS 93-05 and JS 95-60 were repeated and randomized across the blocks. Disease evaluation was done in terms of PDI at R_7_ (physiological maturity) growth stage^34^, using a disease rating scale 0 to 9^35^ (Table 1). Disease reaction on susceptible checks ranged from susceptible to highly susceptible during both years indicating high-disease pressure at the experimental field site.

**Table 1:** Disease rating scale for evaluating soybean genotypes against charcoal rot disease.

Rating	Descriptions	Categories
0	Percent disease incidence	Highly resistant (HR)
1	> 0 to 1% Disease Incidence	Resistant (R)
3	> 1 to 10% Disease Incidence	Moderately resistant (MR)
5	> 10 to 25% Disease Incidence	Moderately susceptible (MS)
7	> 25 to 50% Disease Incidence	Susceptible (S)
9	> 50% Disease Incidence	Highly susceptible (HS)

### Selective screening for charcoal rot resistance

Out of 151 genotypes evaluated during 2019, 43 genotypes were selected based on disease reaction and superior agronomic performance for their further field evaluation and molecular characterization. During 2020 and 2021, these 43 genotypes, along with five checks- JS 20-29, JS 335, JS 93-05, JS 95-60 and Dsb 21 were evaluated for PDI, AUDPC, and grain yield per plot (3.0 × 0.6 m^2^). The experiments were conducted in a RCBD design with three replications. In order to ensure high-disease pressure and no disease escape, seeds were mixed sorghum grain infected with *M. phaseolina* (10 g/each genotype/each replication) before sowing. Prior to mass multiplication of the pathogen, pathogenicity of the isolate (Fig. S1) was confirmed through a cut-stem inoculation technique^36^. Percent Disease Index was recorded during pod development, seed filling and at physiological maturity (between R_6_and R_7_ growth stage) by recording the number of dead plants in each plot. To evaluate the genotypes based on AUDPC, progressive development of disease was recorded at reproductive stages of soybean at 45, 60, 75 and 90 days after sowing.

AUDPC was calculated as per^37^

$${\text{UDPC }} =^{{{\text{n}} - {1}}} \sum_{{{\text{i}} = {1}}} \left[ {\left( {{\text{y}}_{{\text{i}}} + {\text{ y}}_{{{\text{i}} + {1}}} } \right)/{2}} \right]\left[ {{\text{t}}_{{{\text{i}} + {1}}} - {\text{t}}_{{\text{i}}} } \right]$$

where, *y*_*i*_ = per cent incidence of charcoal rot at *i*th observation, *t*_i_ = time (days) at *i*th observation, and n = number of observation

### Diversity analysis of soybean genotypes

Diversity analysis of48 soybean genotypes under study were performed using SSR markers developed by^38^ (table). Plant genomic DNA was extracted using CTAB method (Cetyltrimethylammonium bromide)^39^. The purified plant genomic DNA was quantified using the nanodrop (Denovix DS-11 + spectrophotometer) and the quality of the DNA was checked on 0.8% agarose gel electrophoresis. Polymorphism among the genotypes was determined using 59 SSR markers distributed across the 20 soybean linkage groups (https://soybase.org/). For marker analysis, the purified genomic DNA was subjected to amplification using PCR in reaction mixture (10 µl) containing 1.0 µl DNA (50–70 ng/µl), 1 µl 10 × PCR master mix, 0.6 µl each forward and reverse SSR primers (100 ng/µl), and 6.8 µl molecular-grade water. Amplification using SSR markers was carried out in thermocycler (Applied Biosystems, USA) using the standard protocol conditions with initial denaturation at 94 °C for 5 min, denaturation (94 °C) for 40 s, annealing (55 °C) for 1 min, extension (72 °C) for 1 min and final extension (72 °C) for 7 min. Amplified SSR products were resolved on 3.5% Metaphor agarose (Lonza, Switzerland) and SSR sizes were estimated using 50 bp DNA ladder. All the polymorphic markers in each genotype were recorded for the number of alleles present in the particular marker. Bands were scored as 1 (presence) or 0 (absence) for each allele and missing bands were scored as 9. Polymorphic information content (PIC) and expected heterozygosity (H) values show the discriminating ability of the marker based on the number of known alleles and their frequency distribution. PIC value for each marker was analyzed using the formula given by^40^.

$${\text{PIC }} = {1} - \sum {\text{Pi}}^{{2}}$$

where, Pi indicates the frequency of the ith allele among the genotypes analyzed and was calculated for each SSR locus. Jaccard’s similarity coefficient was employed in estimating the genetic similarity among genotypes. The resulting similarity matrix was further analyzed using the unweighted pair-group method arithmetic average (UPGMA) clustering algorithm for construction of dendrogram.

### Statistical analyses

Prior to analysis, data for Percent Disease Incidence (PDI) was transformed using arcsine transformation^41^ to make the residual normal. Analysis of Augmented Randomized Complete Block Design was carried out using R package “augmentedRCBD”^42^. The Least Significance Difference (LSD) test was carried out using the R package “agricolae”^43^. Analysis of variance, estimation of variance components and heritability and MGIDI analysis was done through R package “metan”^27^. A phylogenetic tree was constructed from genotypic data of polymorphic SSR markers using NTSYSpc version 2.2^44^, on the basis of genetic distances.

### Bioethical statement

We confirmed that all local, national or international guidelines and legislation were adhered for the use of plants in this study (https://www.nature.com/srep/journal-policies/editorial-policies#research-involving-plants).

## Results

### Large-scale screening of soybean germplasm for charcoal rots resistance under sick-plot conditions

During 2018, a total of 230 soybean germplasm lines (including four checks-JS 20-29, JS 335, JS 93-05 and JS 95-60) were screened for charcoal rot resistance under hot-spot conditions. Of them,26 genotypes were highly resistant (HR), 28 genotypes were resistant (R), 36 genotypes were moderately resistant (MR), 41 were moderately susceptible (MS), 43 were susceptible (S) and 56 were highly susceptible (HS). (Table S1 and Figs. S2–S5). Out of 230 soybean genotypes, based on disease reaction and other agronomic traits, 155 genotypes (including four checks-JS 20-29, JS 335, JS 93-05 and JS 95-60)were selected for evaluation during 2019. Of them, 36 genotypes were highly resistant (HR), 21 genotypes were resistant (R), 22 genotypes were moderately resistant (MR), 41 were moderately susceptible (MS), 21 were susceptible (S) and 14 were highly susceptible (HS) (Table S2 and Fig. S1). The ANOVA, showed a significant genotypic effect (*p* < 0.001) for the PDI, during both the years (Table S3).The LSD test revealed that the genotypes significantly varied from each other (*p* < 0.05) for PDI, during both the years of experimentation. (Table S2).

### Evaluation of selected soybean genotypes for PDI, AUDPC and grain yield under high-disease pressure conditions

**Figure 1:**
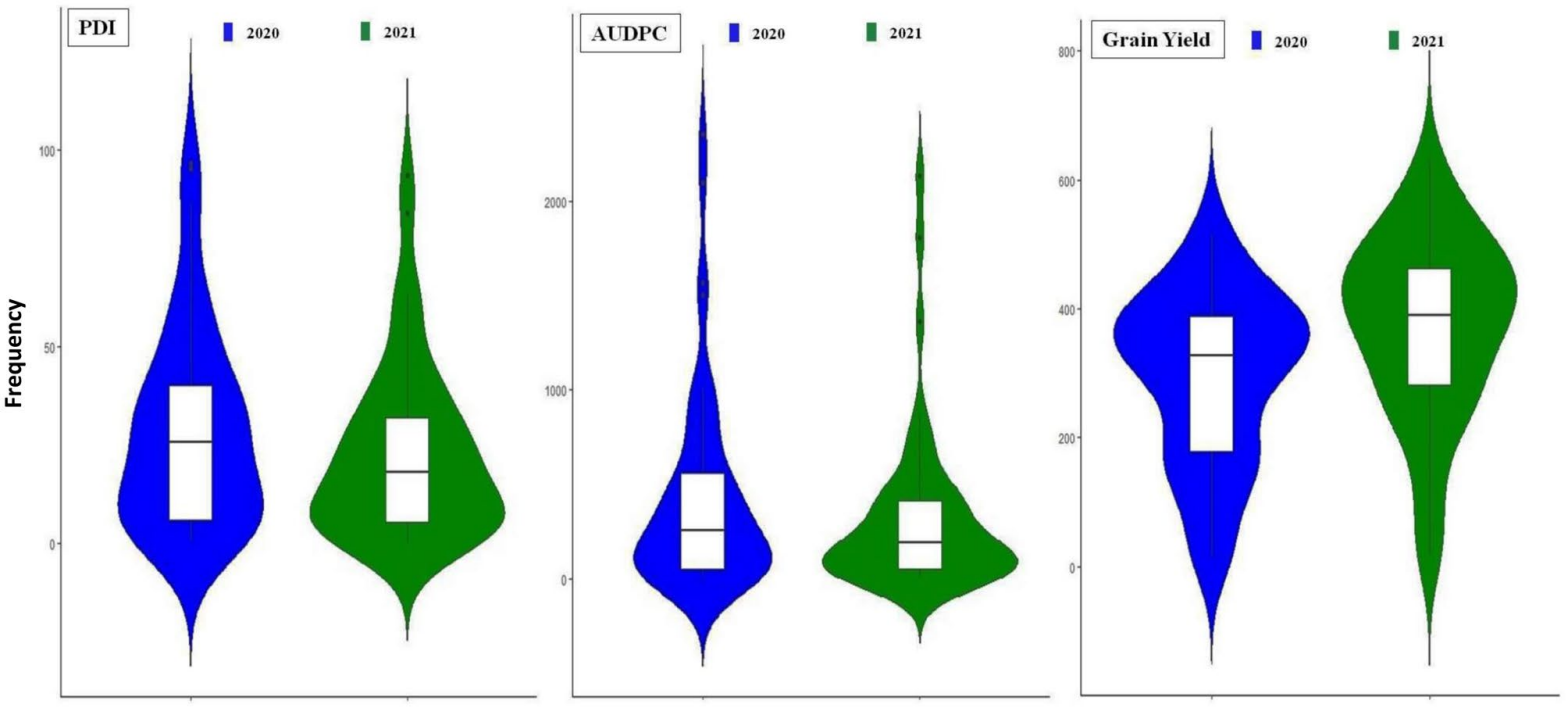
Violin plots for PDI, AUDPC and Grain yield during 2020 and 2021.

Based on disease reaction and other agronomic traits, a total of 43 genotypes were selected from the 2019 experiment. The 43 genotypes, along with five susceptible checks (Dsb 21, JS 95-60, JS 93-05, JS 335 and JS 20-29) were evaluated for PDI, AUDPC and grain yield during 2020 and 2021. Violin plots for different traits during 2020 and 2021 are shown in Fig. 1.

During 2020, PDI ranged from 2.14 (JS 20-20) to 81.67 (JS 95-60), with an average of 30.20. AUDPC ranged from 9.37 (JS 20-20) to 2359.37 (JS 95-60) with a mean of 435.00. The range in grain yield was 15.00 g (DSb 21) to 515.00 g (JS 21-05) with a mean grain yield of 294.00 g. Genotype JS 20-20 had least PDI (0.42) followed by JS 21-05 (1.67), JS 22-01 (1.67) and JS 20-19 (2.50). Genotype JS 20-20 exhibited least AUDPC (9.37) followed by JS 21-05 (18.75), JS 22-01 (18.75) and JS 20-98 (21.87). Highest grain yield was recorded in the genotype JS 21-05 (515.00 g) followed by JS 20-98 (511.67), JS 22-01 (501.67) and JS 21-73 (451.67 g) (Table 2).

**Table 2:** Mean performance of selected genotypes with respect to PDI, AUDPC and Grain yield.

S. no.	Genotype	2020			2021			Pooled		
		PDI	AUDPC	Yield (g/plot)	PDI	AUDPC	Yield (g/plot)	PDI	AUDPC	Yield (g/plot)
1	Dsb 21	95.00^a^ (79.44)	2100.00^b^	15.00^v^	83.75^b^ (66.30)	1809.37^b^	26.67^v^	89.38^a^ (72.87)	1954.68^b^	20.83^uv^
2	NRC 86	30.83^h–k^ (33.71)	300.00^lm^	338.33^g–j^	18.33^m^ (25.32)	218.75^mn^	438.33^j–l^	4.58^k–m^ (29.51)	259.37^l–o^	388.33^f–j^
3	JS 97-52	41.67^fg^ (40.19)	556.25^h^	346.67^g–i^	27.50^i–k^ (31.60)	350.00^l^	408.33^m^	34.58^g–i^ (35.89)	453.12^h–k^	377.50^g–k^
4	AMS 264	7.92^op^ (16.14)	96.87^q–u^	395.00^de^	5.00^st^ (12.85)	37.50^u–x^	511.67^de^	6.46^r–u^ (14.49)	67.18^pq^	453.33^d–f^
5	NRC 128	58.75^d^ (50.05)	953.12^d^	156.67^q–s^	38.33^ef^ (38.24)	443.75^hi^	321.67^o^	48.54^ cd^ (44.14)	698.44f.	239.16^o–s^
6	JS 20-96	4.17^o–r^ (11.58)	37.50^u–w^	461.67^b^	2.08^uv^ (6.75)	21.87^v–x^	443.33^i–k^	3.13^s–u^ (9.16)	29.68^q^	452.50^d–f^
7	PS 1225	14.58^1n^ (22.40)	109.37^p–t^	383.33^ef^	7.08^p–s^ (15.34)	78.12^q–u^	446.67^i–k^	10.83 ^o–s^ (18.87)	93.75^pq^	415.00^e–i^
8	NRC 2755	60.00^d^ (50.78)	1012.50^d^	100.00^u^	48.33^d^ (44.043)	712.50^ef^	208.33^r^	54.17^c^ (47.41)	862.50^e^	154.16^t^
9	SL 955	14.17^n^ (22.03)	106.25^q–u^	386.67^d–f^	10.00^o–q^ (18.37)	106.25^p–s^	486.67^f–g^	12.08^n–r^ (20.20)	106.25^o–q^	436.67^d–g^
10	JS 335	30.83^h–k^ (33.71)	231.25^mn^	270.00^n^	27.92^h–k^ (31.87)	403.12^i–l^	415.00^m^	29.38^h–k^ (32.79)	317.18^k–m^	342.50^j–m^
11	JS 95-60	96.67^a^ (81.67)	2359.37^a^	18.33^v^	93.33^a^ (75.52)	2137.50^a^	16.67^v^	95.00^a^ (78.59)	2248.44^a^	17.50^v^
12	JS 20-19	2.50^q–s^ (8.89)	43.75^t–w^	320.00^i–l^	4.17^st^ (11.75)	50.00^t–x^	365.00^n^	3.33^s–u^ (6.53)	46.87^pq^	342.50^j–m^
13	PS 1641	5.83^opq^ (13.85)	43.75^t–w^	423.33^c^	9.58 ^o–r^ (17.97)	103.12^p–t^	366.67^n^	7.71^q–u^ (15.91)	73.44^pq^	395.00^f–j^
14	CAT 87	33.33^ghij^ (35.23)	568.75^h^	226.67^o^	20.83^kl^ (27.13)	212.50^mn^	371.67^n^	27.08^ijkl^ (31.18)	390.62^i–l^	299.17^l–o^
15	AMS MB-5-18	30.00^ijk^ (33.19)	350.00^kl^	351.67^gh^	42.92^de^ (40.92)	659.37^f^	285.00^p^	36.46^fgh^ (37.05)	504.68^g–i^	318.33^k–n^
16	JS 20-73	5.42^opq^ (13.33)	40.62^t–w^	391.67^de^	5.00^st^ (12.49)	37.50^u–x^	371.67^n^	5.21^rstu^ (12.91)	39.06^q^	381.67^g–k^
17	JS 21-71	5.42^opqr^ (13.16)	53.12^s–w^	390.00^de^	4.58^st^ (12.17)	46.87^u–x^	476.67^f–h^	5.00^rstu^ (12.66)	50.00^pq^	433.33^d–g^
18	CAT 492	52.92^de^ (46.68)	846.87^e^	125.00^tu^	36.67^efg^ (37.24)	431.25^ij^	231.67^r^	44.79^de^ (44.79)	639.06^fg^	178.33^st^
19	JS 21-05	1.67^st^ (6.06)	18.750^vw^	515.00^a^	0.42^vw^ (2.14)	9.37^wx^	598.33^b^	1.04^u^ (1.04)	14.06^q^	556.67^ab^
20	JS 20-53	3.75^pqrs^ (11.06)	53.12^s–w^	331.67^h–k^	3.33^tu^ (8.61)	43.75^u–x^	458.33^h–j^	3.54^stu^ (9.83)	48.44^pq^	395.00^f–j^
21	JS 20-20	0.42^t^ (2.14)	9.37^w^	388.33^d–f^	0.00^w^ (0.00)	0.00^x^	431.67^k–m^	0.21^u^ (1.07)	4.68^q^	410.00^e–i^
22	CAT 1957	56.25^d^ (48.59)	1009.37^d^	150.00^r–t^	29.58^ghij^ (32.93)	365.62^kl^	228.33^r^	42.92^def^ (40.76)	687.50^f^	189.17^r–t^
23	JS 20-30	40.83^fg^ (39.71)	731.25f.	160.00^qr^	22.92^jkl^ (28.58)	215.62^mn^	260.00^q^	31.88^hijk^ (34.14)	473.44^h–j^	210.00^q–t^
24	JS 21-77	31.25^hijk^ (33.95)	428.12^ij^	380.00^ef^	25.83^ijk^ (30.48)	381.25^j–l^	353.33^n^	28.54^hijk^ (32.21)	404.68^i–l^	366.67^h–k^
25	MACS 1370	39.17^fgh^ (38.73)	412.50^ijk^	175.00^p–r^	41.67^de^ (40.20)	450.00^hi^	286.67^p^	40.42^efg^ (39.46)	431.25^h–k^	230.83^p–s^
26	HARDER	44.58^ef^ (41.88)	378.12^jk^	178.33^pq^	22.08^kl^ (28.00)	165.62^no^	371.67^n^	33.33^ghij^ (34.94)	271.87^l–n^	275.00^n–q^
27	JS 21-73	8.33 ^o^ (16.71)	81.25^r–v^	451.67^b^	11.67^nop^ (19.93)	125.00^o–q^	533.33^cd^	10.00^qrst^ (18.32)	103.12^pq^	492.50^b–d^
28	JS 20-98	2.92^rs^ (8.03)	21.87^vw^	511.67^a^	2.08^uv^ (6.75)	15.62^v–x^	631.67^a^	2.50^tu^ (7.39)	18.75^q^	571.67^a^
29	JS 20-29	86.67^b^ (68.77)	1506.25^c^	41.67^v^	62.50^c^ (52.26)	881.25^d^	130.00^t^	74.58^b^ (60.51)	1193.75^d^	85.83^u^
30	MACS 1520	24.17^klm^ (29.40)	275.00^m^	308.33^k–m^	36.25^efg^ (37.00)	409.37^i–k^	355.00^n^	30.21^hijk^ (33.2)	342.18^j–m^	331.67^j–n^
31	EC 393,228	36.67^fghi^ (37.25)	450.00^i^	191.67^p^	57.50^c^ (49.33)	718.75^e^	161.67^s^	47.08^cde^ (43.29)	584.37^f–h^	176.67^st^
32	KDS 1097	28.33^ijk^ (32.13)	406.25^i–k^	313.33^j–m^	11.25^op^ (19.52)	140.62^op^	520.00^de^	19.79^lmn^ (25.82)	273.44^l–n^	416.67^e–i^
33	JS 20-39	4.58^opqr^ (12.27)	53.12^s–w^	383.33^ef^	6.25^qrs^ (14.07)	84.37^q–u^	453.33^h–k^	5.42^rstu^ (13.17)	68.75^pq^	418.33^e–i^
34	JS 93-05	75.00^c^ (60.04)	1568.75^c^	28.33^v^	63.33^c^ (52.75)	1362.50^c^	80.00^u^	69.17^b^ (56.39)	1465.62^c^	54.17^uv^
35	PS 1613	39.58^fgh^ (38.98)	659.37^g^	286.67^mn^	23.33^ijkl^ (28.87)	256.25^m^	466.67^g–i^	31.46^hijk^ (33.92)	457.81^h–k^	376.67^g–k^
36	EC 350,664	30.83^hijk^ (33.72)	462.50^i^	176.67^p–r^	18.75^lm^ (25.59)	240.62^m^	265.00^pq^	24.79^klm^ (29.65)	351.56^i–m^	220.83^p–s^
37	DS 1318	5.00^opqr^ (12.85)	43.75^t–w^	351.67^gh^	5.42^rst^ (13.37)	40.62^u–x^	498.33^ef^	5.21^rstu^ (13.11)	42.18^q^	425.00^e–h^
38	EC 34,117	14.17^n^ (22.03)	150.00^o–r^	291.67^mn^	22.08^kl^ (28.00)	246.87^m^	270.00^pq^	18.13^mnop^ (25.01)	198.44^m–p^	280.83^m–p^
39	PK 768	6.67 (14.87^op^)	56.25^s–w^	403.33^c–e^	6.25^qrs^ (14.43)	65.62^r–v^	461.67^h–j^	6.46^rstu^ (14.65)	60.98^pq^	432.50^d–h^
40	AMS 100-39	33.75^ghij^ (35.50)	440.62^ij^	296.67^mn^	17.92^lm^ (24.96)	221.87^m^	418.33^lm^	25.83^jklm^ (30.23)	331.25^j–m^	357.50^i–l^
41	JSM 283	27.08^jkl^ (31.32)	346.87^kl^	231.67^o^	35.42^efgh^ (36.50)	509.37^g^	265.00^pq^	31.25^hijk^ (33.91)	428.12^i–k^	248.33^o–r^
42	JSM 228	5.83^opq^ (13.85)	43.75^t–w^	361.67^fg^	3.75^stu^ (11.06)	59.37^s–w^	408.33^m^	4.79^rstu^ (12.45)	51.56^pq^	385.00^g–j^
43	JS 22-01	1.67^st^ (6.06)	18.75^vw^	501.67^a^	0.83^vw^ (3.03)	6.25^wx^	580.00^b^	1.25^u^ (4.54)	12.50^q^	540.83^a–c^
44	NRC 138	57.92^d^ (49.56)	815.62^e^	131.67^st^	30.83^fghi^ (33.71)	487.50^gh^	321.67^o^	44.38^def^ (41.63)	651.56^fg^	226.67^p–s^
45	DS 3106	16.67^n^ (24.05)	162.50^n–q^	411.67^cd^	5.42^rst^ (13.16)	40.62^u–x^	535.00^cd^	11.04^opqrs^ (18.60)	101.56^pq^	473.33^de^
46	DS 3104	17.92^mn^ (25.00)	184.37^no^	321.67^i–l^	13.33^mno^ (21.29)	118.75^o–r^	453.33^h–k^	15.63^nopq^ (23.14)	151.56^n–q^	387.50^f–j^
47	CAT 1847	19.58^mn^ (26.20)	178.12^n–p^	340.00^g–j^	17.50^lmn^ (24.67)	131.25^o–q^	365.00^n^	18.54^mno^ (25.43)	154.68^n–q^	352.50^i–l^
48	PS 1611	15.83^n^ (23.38)	118.75^o–s^	403.33^c–e^	4.58^st^ (12.05)	34.37^u–x^	548.33^c^	10.21^pqrst^ (17.71)	76.56^pq^	475.83^c–e^
Mean		30.2	435.00	294.00	25.80	327.00	373.00	28.00	381.00	333.5

Values with the same superscript alphabets are not significantly different.

Figures in parenthesis are arcsine transformed values.

*PDI* Percent Disease Incidence, *AUDPC* Area Under Disease Progress Curve.

During 2021, PDI ranged from 0.00 (JS 20-20) to 75.52 (JS 95-60), with an average of 25.8.AUDPC ranged from 0.00 (JS 20-20) to 2137.50 (JS 95-60) with a mean of 327.00. Grain yield ranged from 16.66 g (JS 95-60) to 631.66 g (JS 20-98) with a mean grain yield of 373.00 g. Genotype JS 20-20 had the lowest PDI (0.00) followed by JS 21-05 (0.42), JS 22-01 (0.83), JS 20-96 (2.08) and JS 20-98 (2.06). Lowest AUDPC was observed in JS 20-20 (0.00) followed by JS 22-01 (6.25), JS 21-05 (9.37) and JS 20-98 (15.62). Highest grain yield was recorded by JS 20-98 (631.66 g) followed by JS 22-01 (580.00 g), PS 1611 (548.33 g) and DS 3106 (535.00 g) (Table 2).

Pooled means across both the years for PDI, AUDPC and grain yield were 28.0, 381.0 and 17.5 g, respectively. PDI, AUDPC and grain yield ranged from 0.20 (JS 20-20) to 95.00 (JS 95-60), 4.68 (JS 20-20) to 2248.43 (JS 95-60) and 17.5 g (JS 95-60) to 571.66 g (JS 20-98), respectively. PDI was least in JS 20-20 (0.21) followed by JS 21-05 (1.04), JS 22-01 (1.25) and JS 20-98 (2.50). The lowest AUDPC was observed in JS 20-20 (4.68) followed by JS 22-01 (12.50), JS 21-05 (14.06) and JS 20-98 (18.75). Grain yield was highest in JS 20-98 (571.67 g) followed by JS 21-05 (556.67 g), JS 22-01 (540.83 g) and JS 21-73 (492.50 g) (Table 2).

#### ANOVA, LRT, variance components and genetic parameters for the traits under study

The ANOVA in each year for 2020 and 2021 revealed that the genotypic effects for the three traits under study were significant at *p* < 0.001 (Table S4. Pooled analysis of variance (ANOVA) across years indicated that the genotypic effect, environmental effect and G × E interaction effect werehighly significant (*p* < 0.001) (Table 3) for the three traits.The genotypic effect for AUDPC contributed 93.6% to the total variation, followed by G × E interaction effect (4.6%) and environmental effect (1.22%). The largest portion of the total variation for PDI was explained by the genotypic effect (92.72%) followed by G × E interaction effect (3.59%) and environmental effect (1.56%). Similarly, 86.22% of the total variation for grain yield was governed by genotypic effect followed by G × E interaction effect (5.29%) and environmental effect (0.07%). Likelihood Ratio Test (LRT) revealed highly significant genotype and G × E interaction effect (*p* < 0.001) for all the three traits under study (data not shown).

**Table 3:** Pooled ANOVA of PDI, AUDPC and Grain yield in selected soybean genotypes evaluated.

Source of variation	DF	AUDPC		PDI		Grain Yield	
		SS	F_cal_	SS	F_cal_	SS	F_cal_
ENV	1	847,032 (1.22%)	546.01***	1391.3 (1.56%)	144.75***	447,300 (0.07%)	1652.68***
REP(ENV)	4	8766 (0.01%)	1.41^NS^	84.3 (0.09%)	2.19^NS^	1017 (0.01%)	0.94^NS^
GEN	47	64,625,731 (93.60%)	886.36***	82,613.5 (92.72%)	182.87***	5,071,713 (86.22%)	398.70***
G × E	47	3,238,710 (4.60%)	44.42***	3200.1 (3.59%)	7.08***	311,375 (5.29%)	24.48***
Error	188	291,644 (0.42%)	-	1807.0 (2.02%)	–	50,883 (0.86%)	–

*NS* non-significant

***Significant at *p* < 0.001.

Different variance components and genetic parameters of the traits under study across both the years are presented in Table S5. During both the years, for all three traits, genotypic variance was higher than the environmental variance. Heritability estimates were high for all the traits under study. Genotypic coefficient of variation (CV_g_) was high for all the three traits, in both the years. Residual coefficients of variation (CV_r_) was medium for PDI and AUDPC and low for grain yield, in both the years.

#### Genotypic BLUP values for PDI, AUDPC and Grain yield

Genotypic BLUP values for the traits PDI, AUDPC and Grain yield across two years are shown in Table S5. During 2020, BLUP value of AUDPC was least in JS 20-20 (10.3) followed by JS 21-05 (19.7), JS 22-01 (19.7) and JS 20-98 (22.8). The least BLUP value for PDI was recorded in JS 20-20 (2.4) followed by JS 21-05 (6.3), JS 22-01 (6.3) and JS 20-98 (8.3). The top four genotypes for grain yield were JS 21-05 (513.7 g), JS 20-98 (510.4 g), JS 22-01 (500.5 g) and JS 20-96 (460.7 g). Likewise, during 2021, the least BLUP value of AUDPC was recorded in JS 20-20 (0.70) followed by JS 22-01 (6.9), JS 21-05 (10.00) and JS 20-98 (16.3). The least BLUP value for PDI was observed in JS 20-20 (0.30) followed by JS 21-05 (2.40), JS 22-01 (3.30) and JS 20-96 (7.00). The grain yield BLUP was highest in the genotype JS 20-98 (630.7 g) followed by JS 21-05 (597.5 g), JS 22-01 (579.20 g) and PS 1611 (547.7 g) (Table S6).

#### Identification of ideotypes using MGIDI index

**Figure 2:**
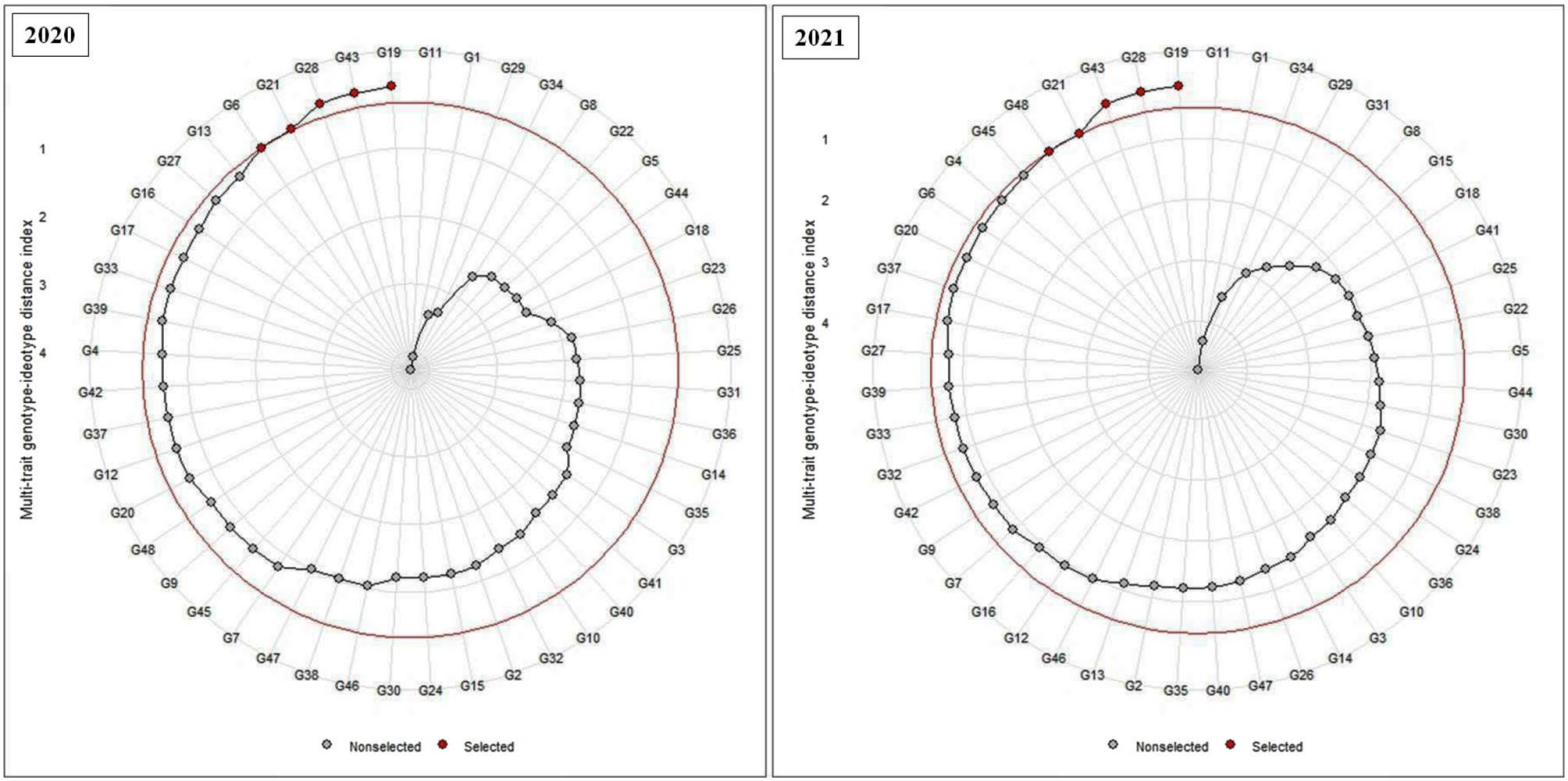
MGIDI index analysis for the 48 genotypes under study.

Using MGIDI index at a 10% selection intensity, genotypic selection was carried out based on multiple traits simultaneously (Table 3). A lower value was desirable for AUDPC and PDI and a higher value was desirable for grain yield. Across both the years, percentage selection differential was highest for AUDPC (94.9% during 2020 and 95.8% in 2021), followed by PDI (76.8% in 2020 and 80.5% in 2021) and grain yield (61.4% in 2020 and 49.4% in 2021). In 2020, JS 21-05 (G19), JS 22-01 (G43), JS 20-98 (G28), JS 20-20 (G21) and JS 20-96 (G6) were identified as ideotypes. Likewise, in 2021, JS 21-05 (G19), JS 20-98 (G28), JS 22-01 (G43), JS 20-20 (G21) and PS 1611 (G48) were ideotypes (Table 4 and Fig. 2). Across both the years, JS 21-05 (G19), JS 22-01 (G43), JS 20-98 (G28) and JS 20-20 (G21) were identified to be common ideotypes with respect to the traits that were evaluated.

**Table 4:** Predicted genetic gains for the traits PDI (Percent Disease Incidence), AUDPC (Area Under Disease Progress Curve) and Grain yield across the years 2020 and 2021 using MGIDI index.

Factor	Trait	Xo	Xs	SD	SDperc	Goal
Year 2020						
FA1	AUDPC	435	22.2	−413	−94.9	Decrease
FA1	PDI	30.2	7.01	−23.2	−76.8	Decrease
FA1	Grain yield	294	475	180	61.4	Increase
Year 2021						
FA1	AUDPC	327	13.8	−313	−95.8	Decrease
FA1	PDI	25.8	5.03	−20.8	−80.5	Decrease
FA1	Grain yield	373	557	184	49.4	Increase

*Xo* the original population mean, *Xs* the mean of selected genotypes, and *SD and SDperc* the selection differential and selection differential in percentage, respectively.

### Molecular diversity analysis

**Figure 3:**
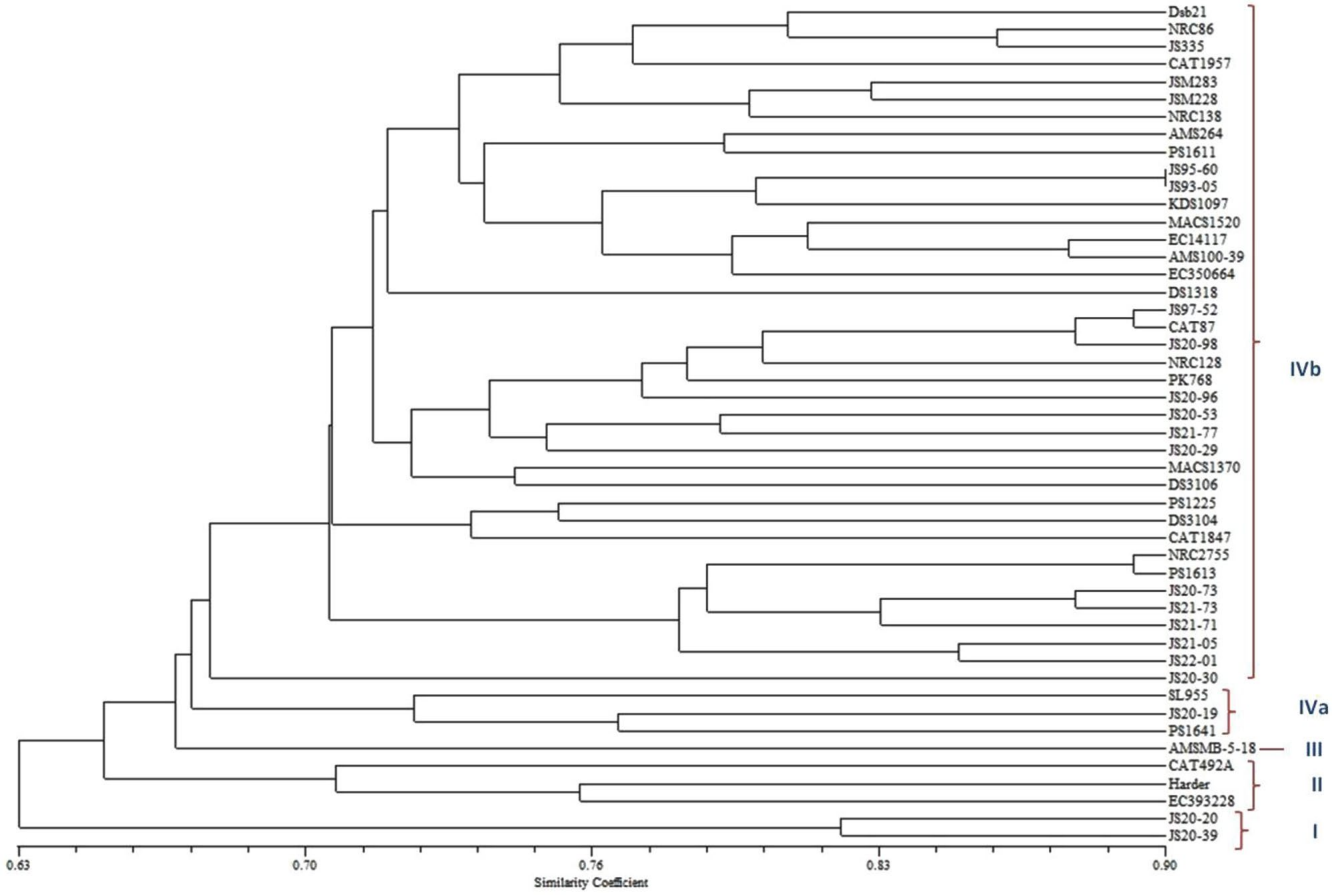
Dendrogram showing genetic relationships among 48 soybean genotypes based on UPGMA clustering of Jaccard’s similarity coefficients.

Fifty-four primers were polymorphic (91.5%) while five primers (Satt155, Satt252, Satt484, Satt575 and Satt724) were monomorphic across genotypes. A total of 142 alleles were amplified with an average of 2.63 alleles per locus. The number of alleles per polymorphic primer pair (locus) ranged from 2 to 6 (Satt373). One locus amplified 5 alleles, 6 loci amplified 4 alleles, 15 loci had 3 alleles and 31 loci had 2 alleles. Out of 142 alleles, 54 alleles had a frequency of 0.25 or less, 22 alleles exhibited a frequency of 0.75 or higher and the remaining 64 alleles had a frequency between 0.25 and 0.75. The size of the allele fragments ranged from 50 to 280 bp. The PIC value, for the 59 SSR markers ranged from 0.00 to 0.737 with 0.357 being the average PIC/ locus (Table 5). Out of 142 alleles identified, 5 alleles (3.5%) were unique and were amplified in a single genotype, they were; Satt 459 (LG D1b & Chromosome no 2) in Dsb1, Satt557 (LG C2 & Chromosome 6) in JS 95-60, Satt588 (LG K & Chromosome 9) in JS 21-71, Sat_218 (LG H & Chromosome 12) in DS 1318 and Satt701 (LG D1b & Chromosome 2) in SL 955. Size of these unique alleles 130, 155, 100, 200 and 150 bp respectively (Table 6). Some of the charcoal rot resistant genotypes viz. JS 21-71and DS 1318 can be identified by the unique alleles based on size of 100 and 200 bp in Satt588 and Sat_218, respectively. All 142 SSR alleles were used for the genetic diversity analysis. Jaccard’s similarity coefficient was calculated to assess the genetic proximity among the genotypes and the similarity coefficient matrix was used for UPGMA cluster analysis. Forty-eight (48) genotypes were grouped into four major clusters. Cluster I contained only two genotypes—JS 20-20 and JS 20-39. JS 20-20 was the most resistant genotype. SSR based diversity analysis separated it in a distinct cluster along with only JS20-39. Therefore, JS 20-20 may be useful for molecular mapping and resistance gene identification studies. Cluster II includes EC 393228, Harder and CAT 492A. Cluster III consists of a single genotype—AMS-MB-5-18. Cluster IV was sub-divided into IVa and IVb. Cluster IVa consists of PS 1641, JS 20-19 and SL 955. The remaining 39 genotypes were included in cluster IVb (Fig. 3).

**Table 5:** Details of 59 SSR markers loci showing number of alleles, PIC value and allele sizes in 48 soybean genotypes.

S.no	Linkage group (chromosome number)	SSR name	Number of alleles	PIC value	Allele size range (bp)
1	D1a (1)	Satt184	2	0.477592	100–120
2	D1a (1)	Satt077	2	0.121928	70–80
3	D1b (2)	Sat_227	2	0.325413	200–210
4	D1b (2)	Satt 459	2	0.040799	130–150
5	N (3)	Sat_195	2	0.079861	100–110
6	N (3)	Satt022	2	0.442274	140–160
7	N (3)	Satt387	2	0.21875	150–155
8	C1 (4)	Satt164	2	0.457465	180–185
9	C1 (4)	Satt396	2	0.494341	130–135
10	A1 (5)	Satt155	Monomorphic	0	–
11	A1 (5)	Satt200	2	0.470349	190–200
12	A1(5)	Satt717	2	0.499132	210–230
13	C2 (6)	Satt252	Monomorphic	0	–
14	C2 (6)	Satt557	4	0.462653	135–155
15	M (7)	Sat_316	3	0.322318	200–250
16	M (7)	Sat_276	3	0.551649	200–270
17	A2 (8)	Satt406	3	0.505642	90–140
18	A2 (8)	Satt480	3	0.414063	135–145
19	K (9)	Satt588	4	0.550123	100–200
20	O (10)	Sat_196	3	0.612949	150–205
21	O (10)	Sat_190	3	0.466667	80–120
22	B1 (11)	BE806308	2	0.249132	140–180
23	B1 (11)	Satt484	Monomorphic	0	–
24	H (12)	Satt666	2	0.186632	190–200
25	H (12)	Sat_218	4	0.594518	200–250
26	F (13)	Sat_390	3	0.535989	200–250
27	F (13)	Satt362	3	0.550781	200–215
28	B2 (14)	Satt126	2	0.470558	70–100
29	B2 (14)	Satt687	3	0.492188	120–130
30	E (15)	Satt411	2	0.105469	50–80
31	E (15)	Satt230	2	0.329861	180–185
32	E (15)	Satt575	Monomorphic	0	–
33	E (15)	Satt384	2	0.152778	70–100
34	E (15)	Satt651	2	0.277778	130–150
35	E (15)	Satt724	Monomorphic	0	–
36	E (15)	Sat_381	3	0.283854	140–180
37	J (16)	Sat_393	2	0.199219	250–280
38	J(16)	Satt244	4	0.482571	100–160
39	J(16)	Satt456	2	0.152778	250–270
40	J(16)	Satt249	2	0.394965	190–200
41	J (16)	Sat_412	3	0.468148	220–250
42	D2 (17)	Satt310	2	0.418289	180–185
43	G(18)	Satt163	2	0.380263	200–250
44	G (18)	Satt517	2	0.309642	220–270
45	G (18)	Satt566	2	0.363894	230–240
46	L (19)	Sat_286	2	0.292346	80–100
47	L (19)	Satt373	6	0.618802	180–250
48	I (20)	Sat_299	2	0.449072	220–250
49	I (20)	Satt270	3	0.45043	150–190
50	D1b(2)	Satt701	4	0.553819	150–180
51	C1 (4)	Satt136	2	0.309642	235–240
52	D2 (17)	Satt447	3	0.259549	210–250
53	M (7)	Sat_244	5	0.736979	150–210
54	A2 (8)	Satt378	2	0.400181	100–110
55	K (9)	Satt167	3	0.543232	200–240
56	O (10)	Satt420	2	0.314745	180–190
57	B1 (11)	Satt519	2	0.084938	190–200
58	H (12)	Satt442	4	0.604799	190–240
59	F (13)	Satt554	3	0.556049	190–220
			142	Avg = 0.357/

**Table 6:** Details of five unique SSR alleles identified in the current study.

S. no	SSR name	Linkage group (Chromosome number)	Unique allele size (bp)	Genotype showing unique allele
1	Satt 459	D1b (2)	130	Dsb1
2	Satt557	C2 (6)	155	JS 95-60
3	Satt588	K (9)	100	JS 21-71
4	Sat_218	H (12)	200	DS 1318
5	Satt701	D1b (2)	150	SL 955

### Structure analysis

**Figure 4:**
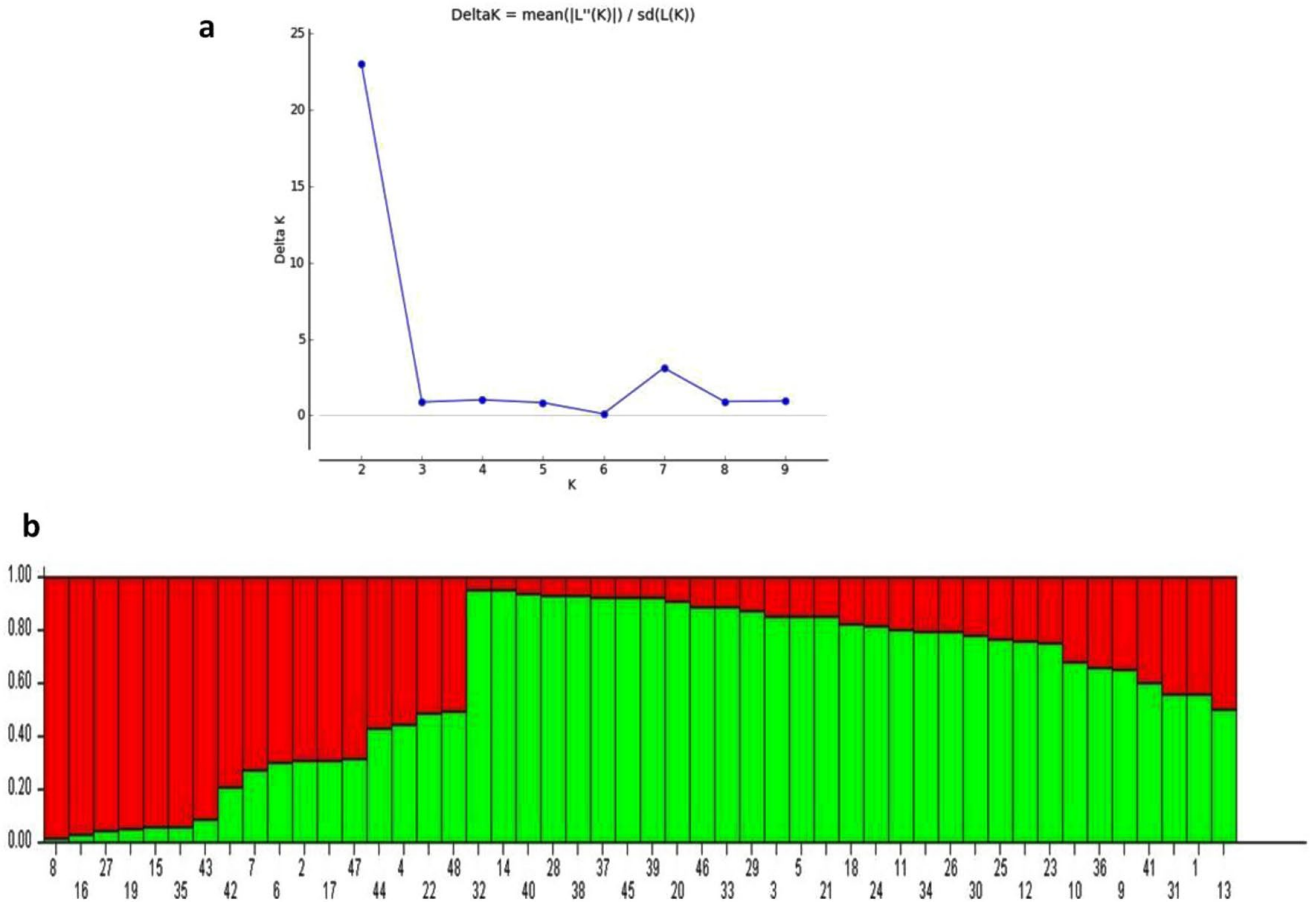
(**a**) Determination of optimum number of genetic clusters (K) using second order statistics (ΔK). (**b**) Population structure of 48 soybean accessions based on SSR genotyping; each accession is represented by a single vertical line and numbers represent soybean accessions as listed in table.

The 59 SSR markers werealso used to study the population structure of the 48 genotypes. A sharp peak in DK at K = 2 suggested the presence of two major populations (Fig. 4a). Population 1 (POP1)contained seventeen genotypes viz., NRC 2755, JS 20-73, JS 71-73, JS 21-05, AMS MB 5-18, PS 1613, JS 22-01, JSM 228, PS 1225, JS 20-96, NRC 86, JS 21-71, CAT 1847, NRC 138, AMS 264, CAT 1957 and PS 1611. The remaining 31 genotypes were grouped into Population 2 (POP2). Overall proportion of membership of the sample in POP1 and POP2 was 0.398 and 0.602, respectively. Average distance (expected heterozygosity) between individuals in same cluster was 0.260 in POP1 and 0.307 in POP2. Mean value of Wrights fixation index (Fst) was 0.273 in POP1 and 0.081 in POP2. Allele-frequency divergence among the two populations was 0.039 (Table 7). Population 1 included the majority of the genotypes bred at Jabalpur (06) followed by genotypes developed at Indore (03), germplasm lines (03), developed at Amravati (02) and Pantnagar (02). Population 2 consisted of the majority of genotypes developed at Jabalpur (13), followed by germplasm lines (05), genotypes developed at New Delhi (03), genotypes developed at Pune (02), Pantnagar (02), Ludhiana (01), KasbeDigraj (01), Amravati (01), Indore (01) and Pantnagar (01) (Fig. 4b).

**Table 7:** Details of membership parameters observed among two populations through structure analysis.

Population	No of genotypes	Overall proportion of membership of the sample	Average distances between individuals in same cluster	Mean value of Fst	Allele-frequency divergence among populations
POP 1	17	0.398	0.260	0.273	0.039
POP 2	31	0.602	0.307	0.081	

*Fst* wrights fixation index.

## Discussion

Though charcoal rot is a major fungal disease in India, to date, no systematic study on identification of high-yielding and charcoal rot resistant genotypes has been carried out. Based on grain yield and resistance reaction, the purpose of current study was identification of high-yielding charcoal rot resistant genotypes and molecular characterization of these genotypes using SSR markers under high-disease pressure.

Above ground charcoal rot symptoms start to appear from R_4_stage (2 cm longpod at one of the four upper most nodes with a completely unrolled leaf). It was observed that the increase in colonization of soybean by *M.phaseolina* was low during the vegetative and early reproductive stages, and reached its peak during R_5_ (beginning of pod development)to R_7_ growth stages^6^.Therefore, in this study, AUDPC was recorded during reproductive stages and PDI was recorded at R_7_ stage, the ideal growth stage to evaluate charcoal rot plant resistance^7^.

Lower levels of residual coefficient of variation (CV_r_) indicate the quality of experimentation. In this study, CV_r_ was lower for grain yield and intermediate for AUDPC and PDI indicating relative uniformity of the disease pressure across the experimental site.Higher genotypic variance and heritability estimates indicate higher response to selection for the traits under this study. The pooled ANOVA revealed that the genotypic variance contributed predominantly to the total variation. The significant G × E interaction effect was observed, indicating that genotypes did not respond the same to the disease across the environments.

Improvement in any economic trait depends on understanding its mode of inheritance and heritability. No extensive studies have been carried out related to soybean charcoal rot resistance^12^. A polygenic mode of inheritance for resistance to *M.phaseolina* in soybean was reported in a few studies [18 and 12]. One QTL on chromosome 15 and two QTL on chromosome 16, governing charcoal rot resistance in soybean was mapped using F_2:3_ derived lines from the cross of PI 567562A (R) × PI 567,437 (S)^12^. In other crops like Sorghum^45^ and common bean^46^ epistatic interactions were observed. Inadequate information on the genetic mechanisms underpinning resistance and significant effects of environment has hindered the progress in breeding for resistance^14^. Nevertheless, transgressive segregation of progeny derived from resistant parents can be useful to identify novel and durable resistant sources^47^. In the current study, based on the pooled data, JS 20-20 was identified as the best genotype for mapping of genes/QTLs governing charcoal rot resistance. JS 20-98 was identified as an appropriate genotype for use as a parent in breeding for higher yield and resistance under high-disease pressure.The use of MGIDI index in selecting ideal genotypeswith resistance based on the multiple traits evaluated in this study, was effective in selecting for yield^48^ and quality traits^49^. In the current study, JS 21-05, JS 22-01, JS 20-98 and JS 20-20 were identified as the ideotypes based on the traits- PDI, AUDPC and grain yield. These genotypes were determined to be potentially high yielding sources with charcoal rot resistance. Hybridization among these ideotypes can result in selection of superior segregants having higher yield and CR resistance.

In addition, traits such as 100-seed weight, plant height, number of nodes, number of branches, biomass and harvest index should be considered in future studies to identify traits associated with yieldand disease indices under high-disease pressure similar to grain mold resistance in sorghum ^50,51^, and fall armyworm resistance in maize^52^.

To assess variation, SSR markers have been widely used for the screening of soybean germplasm^53,54^. In our study, 59 markers distributed uniformly across 20 linkage groups were used for molecular characterization. The high percentage of polymorph ism and high mean PIC value detected in this study is consistent with the previous studies^31,55,56^. However, lower number of alleles per locus indicates a relatively narrow genetic base among the genotypes used in this study.

In the current study, Satt373 had a PIC value of 0.619 with 6 alleles and Sat_244 had 5 alleles with a high PIC value of 0.737. However, satt440 marker with PIC value > 0.6 with the highest number of alleles (4) denotes a strong correlation between PIC value and allele richness. Two unique alleles that can identify different resistant genotypes were identified in this study. Cluster analysis, indicated that majority of genotypes developed at Jabalpur were grouped under a single cluster, IIb2. This indicates the genetic relatedness and narrow genetic base of the genotypes bred at Jabalpur. Except for the AMS-MB-5-18,the remaining genotypes included in POP 1 in structure analysis were included in the cluster IVb, indicating the consistency between cluster analysis and structure analysis for determining genetic relatedness among genotypes evaluated in this study.

In India, apart from charcoal rot disease, Rhizoctonia aerial blight (RAB), YMV and anthracnose are the predominant diseases that can cause significant yield losses. Mega-varieties such as JS 95-60 and JS 93-05 are highly susceptible to all these diseases. Genotypes such as JS 21-71, JS 21-72, JS 21-05, JS 21-17, PS 1611, JS 20-98 and JS20-20 were identified to be resistant to charcoal rot in the current study, were also reported as RAB resistant^57–59^. Genotypes JS 20-98, JS 21-05, JS 21-17 and PS 1611 were reported to be YMV resistant^58,60^, while JS 20-98, PS 1611were reported to be anthracnose resistant^59,61^. These genotypes can be utilized as parents to develop multiple disease resistant varieties that can play a crucial role in enhancing soybean productivity in India.

## Conclusion

In the current study, JS 20-20 was identified as the best genotype for resistance and JS 20-98 was superior in grain yield under high disease pressure. Genotypes JS 21-05, JS 22-01, JS 20-98 and JS 20-20 were identified as ideal ideotypes with respect to AUDPC, PDI and grain yield for charcoal rot resistance. These genotypes will be used as parents to develop high-yielding charcoal rot resistant varieties. Two unique alleles Satt588 (100 bp) and Sat_218 (200 bp) were specific in two resistant genotypes JS 21-71and DS 1318, respectively. In the molecular diversity study, JS20-20 formed a distinct cluster and therefore may be useful in resistance gene mapping and characterization studies. Clustering pattern, showed that the genotypes bred at Jabalpur were genetically more closely related compared to other genotypes.

## Supplementary Information

Supplementary Information.

## Data Availability

All data generated or analyzed during this study are included in this published article and its supplementary information files.
