# Supplementary material for: Identification and genetic diversity analysis of high-yielding charcoal rot resistant soybean genotypes
Source: Sci Rep. 2023 Jun 1;13:8905. doi: 10.1038/s41598-023-35688-2 (PMC10235417; doi:10.1038/s41598-023-35688-2)
Supplement: Supplementary file 1 — Supplementary Information. [file 41598_2023_35688_MOESM1_ESM.docx]

**Figure S1. Ten days old Culture of *Macrophomina phaseolina* in PDA medium (A) and presence of abundant black microslerotia (B)**

**
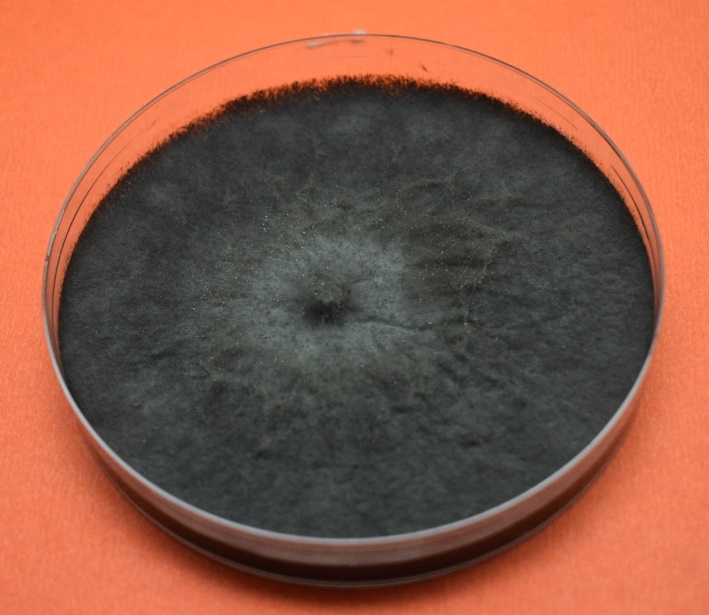

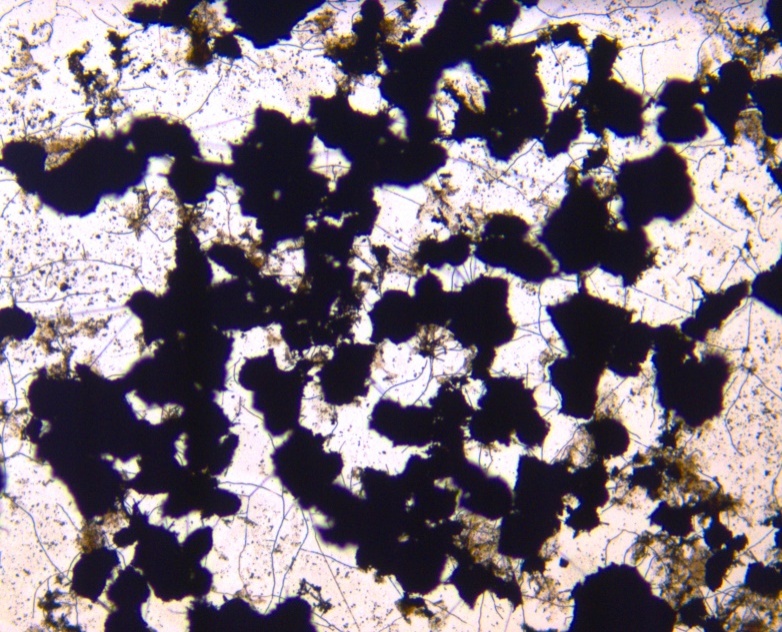
**

**A**

**B**

**Figure S2: Frequency distribution of disease reaction during 2018 and 2019**


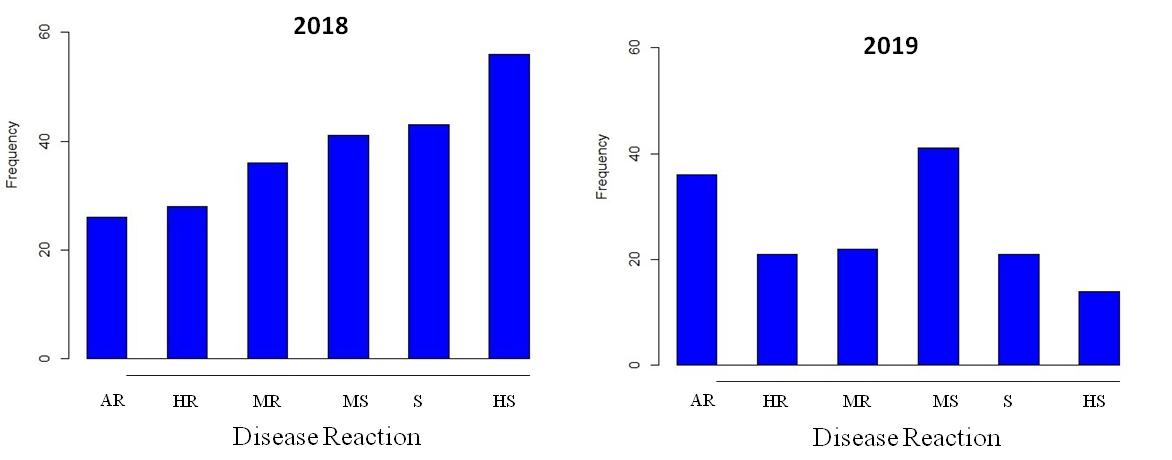


**Figure S3. Charcoal rot affected plants (A) and infected lower stem portion (B)**

**
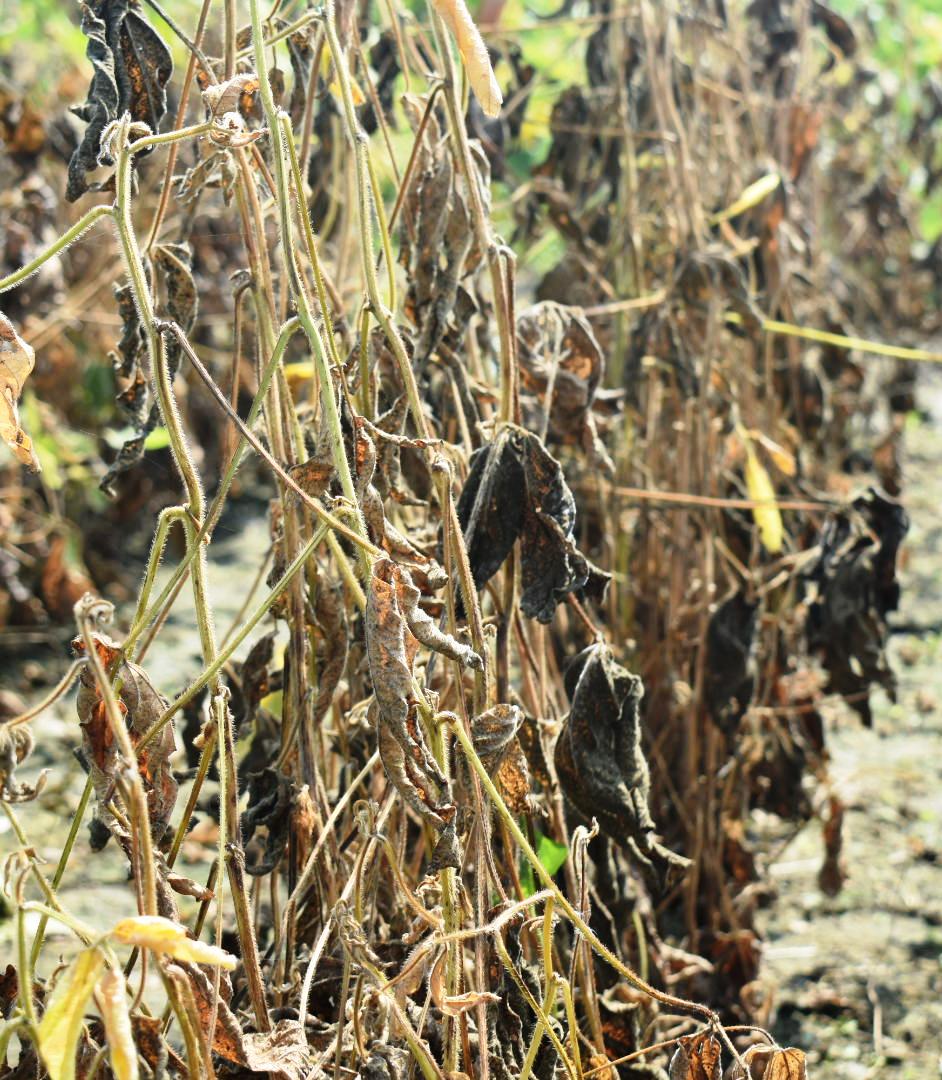

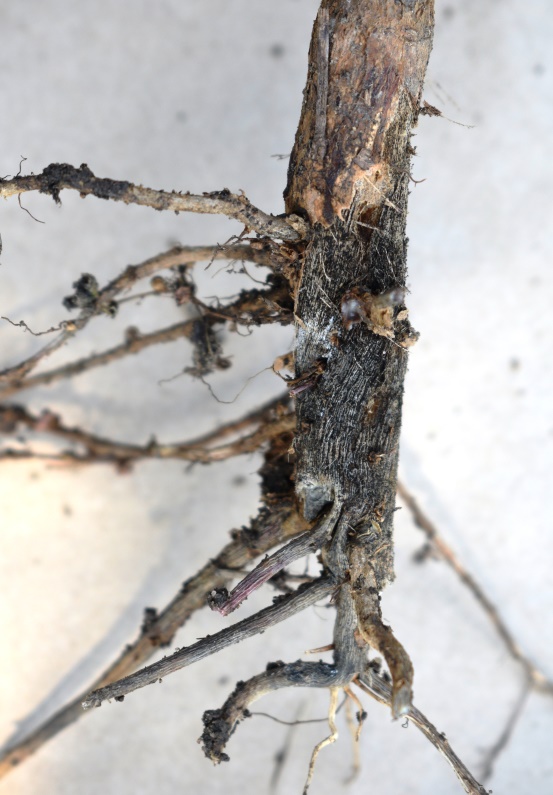
**

**B**

**A**

**Figure S4. Close up view of resistant and susceptible genotypes during evaluation**

**
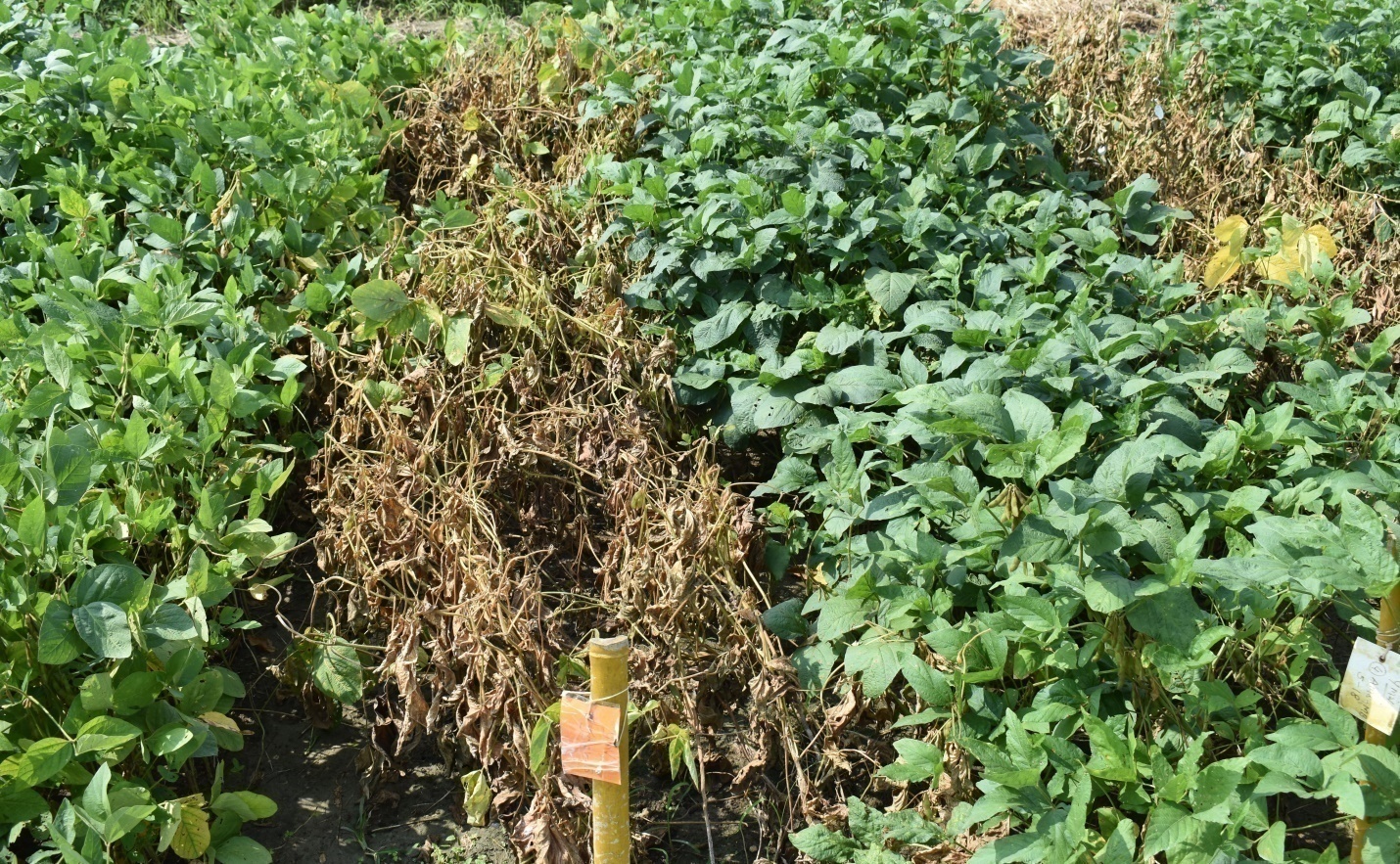
**

**Figure S5. Sick plot showing genotypic variation for charcoal rot incidence in soybean**

**
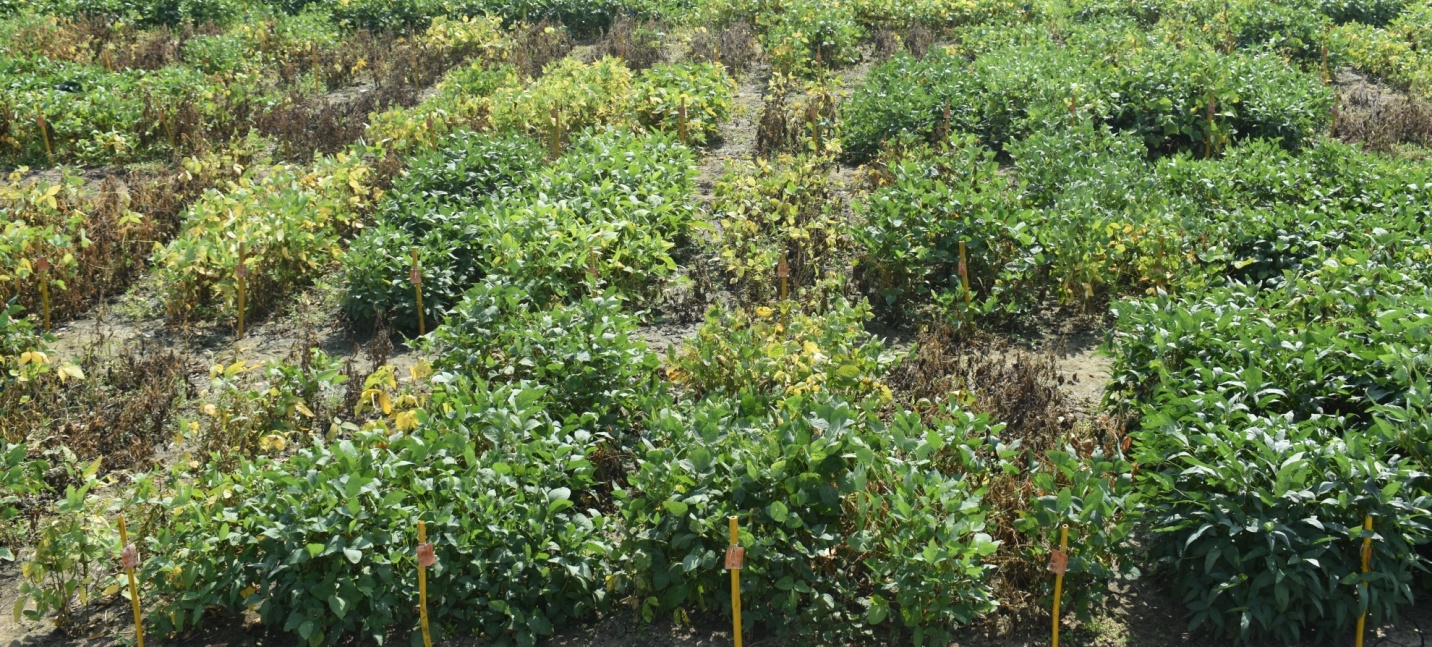
**

**Table S1: Percent Disease Incidence of soybean genotypes during preliminary evaluation in the year 2018. PDI-Percent Disease Incidence, DR-Disease Reaction, HR- Highly Resistant, R- Resistant, MR-Moderately Resistant, MS-Moderately Susceptible, S-Susceptible and HS-Highly Susceptible. Pair-wise comparison using LSD (p < 0.05)**

| S.No | Genotype | PDI | DR | S.No | Genotype | PDI | DR | S.No | Genotype | PDI | DR |
| --- | --- | --- | --- | --- | --- | --- | --- | --- | --- | --- | --- |
| 1 | EC 34117 | 0.001^1^ | R | 79 | AMS 269 | 6.12^1-A^ | MR | 157 | JS 21-03 | 39.20 ^a-u^ | S |
| 2 | AMS 100-39 | 0.001^1^ | R | 80 | AGS-59 | 8.19^1-E^ | MR | 158 | EC 172613 | 39.57 ^a-t^ | S |
| 3 | AMS 264 | 0.001^1^ | R | 81 | AMS 38-24 | 8.19^1-E^ | MR | 159 | AQL 37 | 39.88 ^b-v^ | S |
| 4 | AMS 475 | 0.001^1^ | R | 82 | Cat 1957 | 8.19^1-E^ | MR | 160 | JS 20-76 | 40.83 ^a-u^ | S |
| 5 | AMS MB 5-18 | 0.001^1^ | R | 83 | JSM 229 | 8.69^1-I^ | MR | 161 | SL 958 | 41.78 ^d-w^ | S |
| 6 | B 1664 | 0.001^1^ | R | 84 | EC 241709 | 8.70^1- G^ | MR | 162 | JS 97-57 | 42.12 ^c-w^ | S |
| 7 | B 1667 | 0.001^1^ | R | 85 | EC 389748 | 8.87^1-H^ | MR | 163 | JS 20-37 | 42.27 ^b-w^ | S |
| 8 | Cat 1847 | 0.001^1^ | R | 86 | EC 396055 | 8.87^1-H^ | MR | 164 | EC 287754 | 44.61 ^f-y^ | S |
| 9 | Cat 2126B | 0.001^1^ | R | 87 | JS 21-74 | 9.80^1-J^ | MR | 165 | SQL 36 | 44.68 ^g-x^ | S |
| 10 | Cat 2130 B | 0.001^1^ | R | 88 | G 305 | 9.95^1-J^ | MR | 166 | MACS 1407 | 45.12 ^h-y^ | S |
| 11 | Cat 2310 | 0.001^1^ | R | 89 | AGS 31 | 9.95^1-J^ | MR | 167 | PS 1589 | 46.83 ^j-1^ | S |
| 12 | Cat 2503 | 0.001^1^ | R | 90 | Cat 1368 | 9.95^1-J^ | MR | 168 | EC 291398 | 47.02 ^k-2^ | S |
| 13 | CAT 2511 | 0.001^1^ | R | 91 | DSB 25 | 10.77^1-K^ | MS | 169 | EC 393231 | 47.02 ^k-2^ | S |
| 14 | CAT 87 | 0.00^1-4^ | HR | 92 | NRC SL 2 | 11.37^1-L^ | MS | 170 | JS 20-50 | 47.29 ^k-3^ | S |
| 15 | DS 1318 | 0.00^1-4^ | HR | 93 | JS 21-06 | 11.87^1-M^ | MS | 171 | EC 109540 | 47.47 ^l-5^ | S |
| 16 | DS 3104 | 0.00^1-4^ | HR | 94 | JS 22-09 | 11.87^1-M^ | MS | 172 | Cat 492 | 48.20 ^l-7^ | S |
| 17 | DS 3106 | 0.00^1-4^ | HR | 95 | JS 99-78 | 11.87^1-M^ | MS | 173 | EC 39107 | 48.47 ^l-8^ | S |
| 18 | EC 250608 | 0.00^1-4^ | HR | 96 | JS 20-36 | 13.06^1-N^ | MS | 174 | PS 1569 | 48.98 ^m-9^ | S |
| 19 | EC 350664 | 0.001^12^ | R | 97 | JSM 230 | 13.43^1-M^ | MS | 175 | EC 538830 | 50.68 ^n-0^ | HS |
| 20 | EC 391181 | 0.001^12^ | R | 98 | JS 20-103 | 13.60^1-N^ | MS | 176 | JS 97-52 | 50.75 ^o-A^ | HS |
| 21 | EC 393222 | 0.001^12^ | R | 99 | JS 20-15 | 13.60^1-N^ | MS | 177 | JSM 115 | 50.75 ^o-A^ | HS |
| 22 | EC 393228 | 0.001^12^ | R | 100 | RVS 2007-06 | 13.71^1-N^ | MS | 178 | SL 710 | 53.32 ^p-B^ | HS |
| 23 | EC 547464 | 0.001^12^ | R | 101 | Cat 489 A | 14.08^1-O^ | MS | 179 | JSM 242 | 53.77 ^q-B^ | HS |
| 24 | G -10525 | 0.001^12^ | R | 102 | JS 20-84 | 14.62^1-O^ | MS | 180 | JS 21-77 | 55.19 ^s-E^ | HS |
| 25 | Harder | 0.001^12^ | R | 103 | JS 20-92 | 14.62^1-O^ | MS | 181 | Cat 408 | 55.62 ^s-D^ | HS |
| 26 | JS 20-18 | 0.001^12^ | R | 104 | RVS 2009-9 | 15.47^2-N^ | MS | 182 | EC 468597 | 56.08 ^t-F^ | HS |
| 27 | JS 20-19 | 0.14^1-6^ | R | 105 | JS 20-27 | 16.03^3-Q^ | MS | 183 | ERS 0757 | 56.88 ^t- H^ | HS |
| 28 | JS 20-20 | 0.14^1-6^ | R | 106 | JS 20-56 | 16.03^3-Q^ | MS | 184 | EC 289099 | 57.18 ^u-J^ | HS |
| 29 | JS 20-23 | 0.14^1-6^ | R | 107 | JS 20-71 | 16.03^3-Q^ | MS | 185 | JS 20-48 | 57.72 ^v-K^ | HS |
| 30 | JS 20-39 | 0.14^1-6^ | R | 108 | NRC 138 | 16.75^3-S^ | MS | 186 | JS 20 -41 | 59.39 ^w-N^ | HS |
| 31 | JS 20-53 | 0.14^1-6^ | R | 109 | EC 109543 | 16.81^5-S^ | MS | 187 | EC 241695 | 60.44 ^x-zO^ | HS |
| 32 | JS 20-57 | 0.14^1-6^ | R | 110 | RSC 11-03 | 17.02^3-S^ | MS | 188 | PS 1592 | 60.92 ^y-zP^ | HS |
| 33 | JS 20-73 | 0.14^1-6^ | R | 111 | SKF SPC 11 | 17.02^3-S^ | MS | 189 | JS 20-29 | 61.56 ^z8-zL^ | HS |
| 34 | JS 20-78 | 0.14^1-6^ | R | 112 | JS 20-90 | 17.35^4-S^ | MS | 190 | JS 93-05 | 62.03 ^z9-zL^ | HS |
| 35 | JS 20-96 | 0.14^1-6^ | R | 113 | JS 21-13 | 17.91^6-R^ | MS | 191 | SL 900 | 63.38 ^z2-zQ^ | HS |
| 36 | JS 20-98 | 0.14^1-6^ | R | 114 | JS 21-75 | 17.91^6-R^ | MS | 192 | NRC 117 | 64.82 ^z1-zQ^ | HS |
| 37 | JS 21 -70 | 0.14^1-6^ | R | 115 | JS 99-89 | 17.91^6-R^ | MS | 193 | G 288 | 64.92 ^z3-zQ^ | HS |
| 38 | JS 21-05 | 0.71^1-7^ | R | 116 | JSM 250 | 18.16^5-S^ | MS | 194 | JS 20-79 | 65.41 ^z4-zR^ | HS |
| 39 | JS 21-17 | 0.71^1-7^ | R | 117 | NRC 100 | 18.16^5-S^ | MS | 195 | JSM 245 | 65.70 ^z6-zS^ | HS |
| 40 | JS 21-18 | 0.71^1-7^ | R | 118 | NRC 133 | 18.16^5-S^ | MS | 196 | KDS 256 | 65.70 ^z6-zS^ | HS |
| 41 | JS 21-71 | 0.71^1-7^ | R | 119 | SQL 89 | 18.44^7-T^ | MS | 197 | PANDRINATH | 65.70^z6-zS^ | HS |
| 42 | JS 21-72 | 0.71^1-7^ | R | 120 | VLS 69 | 18.44^7-T^ | MS | 198 | EC 396059 | 65.94 ^z7-zS^ | HS |
| 43 | JS 21-73 | 0.71^1-7^ | R | 121 | NRC 121 | 19.47^0-V^ | MS | 199 | JS 20-74 | 67.35 ^z0-zS^ | HS |
| 44 | JS 21-76 | 0.71^1-7^ | R | 122 | JS 20-40 | 19.74^8-X^ | MS | 200 | JS 20 -51 | 68.11 ^zA-zS^ | HS |
| 45 | JS 22-01 | 0.71^1-7^ | R | 123 | PI 210178 | 19.75^9-Y^ | MS | 201 | JS 20-31 | 68.37 ^z0-zS^ | HS |
| 46 | JS 22-02 | 0.71^1-7^ | R | 124 | AGS-205 | 19.81^8-Z^ | MS | 202 | MACS 1442 | 68.50 ^z0-zS^ | HS |
| 47 | JS 22-03 | 0.71^1-7^ | R | 125 | AGS 48 | 20.80^A-Z^ | MS | 203 | EC 39044 | 69.27 ^zB-zS^ | HS |
| 48 | JS 22-04 | 0.71^1-7^ | R | 126 | AMS 358 | 23.59^B-a^ | MS | 204 | JS 20-49 | 69.44 ^zB-zS^ | HS |
| 49 | JS 22-05 | 0.71^1-7^ | R | 127 | EC 590225 | 24.27^B-e^ | MS | 205 | BRG 1 | 69.82 ^zB-zT^ | HS |
| 50 | JS 22-06 | 0.71^1-7^ | R | 128 | SL 1074 | 24.31^B-c^ | MS | 206 | EC 245988 | 72.40 ^zC-zT^ | HS |
| 51 | JS 22-07 | 0.71^1-7^ | R | 129 | DS 3105 | 24.33^B-e^ | MS | 207 | EC528640 | 73.23 ^zD-zU^ | HS |
| 52 | JS 22-08 | 0.71^1-7^ | R | 130 | AGS174 | 24.48^F-e^ | MS | 208 | EC 280148 | 73.71 ^zF-zU^ | HS |
| 53 | JSM 146 | 0.71^1-7^ | R | 131 | JSM 122 | 24.48^B-d^ | MS | 209 | JS 20- | 74.2555^zG-zU^ | HS |
| 54 | JSM 149 | 0.71^1-7^ | R | 132 | RSC 10-52 | 25.33^B-c^ | S | 210 | EC 33940 | 74.78 ^zI-zU^ | HS |
| 55 | JSM 228 | 2.27^1-9^ | MR | 133 | VLS 94 | 25.33^B-c^ | S | 211 | G 2263 | 74.78 ^zI-zU^ | HS |
| 56 | JSM 265 | 2.27^1-9^ | MR | 134 | EC393224 | 26.02^C -e^ | S | 212 | JS 20-52 | 75.66 ^zM-zU^ | HS |
| 57 | JSM 298 | 2.27^1-9^ | MR | 135 | EC 16213 | 26.17^D -e^ | S | 213 | AMS 148 | 75.86 ^zM-zU^ | HS |
| 58 | JSM 301 | 2.27^1-9^ | MR | 136 | MACS 1543 | 27.00^J-g^ | S | 214 | HARA SOYA | 76.54 ^zO-zU^ | HS |
| 59 | KDS 1009 | 2.27^1-9^ | MR | 137 | NRC 128 | 27.00^J-g^ | S | 215 | SL 599 | 76.65 ^zM-zU^ | HS |
| 60 | KDS 1073 | 2.27^1-9^ | MR | 138 | SL 955 | 28.21^K- h^ | S | 216 | CAT 905 | 76.68 ^zN-zU^ | HS |
| 61 | KDS 1097 | 2.27^1-9^ | MR | 139 | EC 241690 | 28.77^L-i^ | S | 217 | DSb 21 | 76.68 ^zN-zU^ | HS |
| 62 | MACS 1520 | 2.27^1-9^ | MR | 140 | TYPE 49 | 28.83^M-j^ | S | 218 | EC 173325 | 76.68 ^zN-zU^ | HS |
| 63 | MACS 1566 | 2.27^1-9^ | MR | 141 | EC 172663 | 29.61^M-k^ | S | 219 | JS 20-82 | 77.22 ^zM-zU^ | HS |
| 64 | NRC 86 | 2.27^1-9^ | MR | 142 | JSM 283 | 30.59^N-l^ | S | 220 | EAGLE 51 | 78.44 ^zP-zU^ | HS |
| 65 | NRC 99 | 2.27^1-9^ | MR | 143 | RVS 2002-4 | 31.71^O-l^ | S | 221 | JS 95-60 | 78.98 ^zS-zU^ | HS |
| 66 | PI 204336 | 2.55^1-9^ | MR | 144 | WT 88 | 31.71^O-1^ | S | 222 | CAT 539 | 80.51 ^zQ-zU^ | HS |
| 67 | PK 768 | 2.55^1-9^ | MR | 145 | JS 20-30 | 32.56^P-m^ | S | 223 | EC 103336 | 80.51 ^zQ-zU^ | HS |
| 68 | PS 1469 | 2.55^1-9^ | MR | 146 | JS 335 | 32.84^W-i^ | S | 224 | CAT 3284 | 83.19 ^zR-zU^ | HS |
| 69 | PS 1611 | 2.55^1-9^ | MR | 147 | JS 21-78 | 33.92^Q-n^ | S | 225 | SL 744 | 83.45 ^zR-zU^ | HS |
| 70 | PS 1613 | 2.55^1-9^ | MR | 148 | Shivalik | 34.05^Q-o^ | S | 226 | EC 308287 | 87.70 ^zTzU^ | HS |
| 71 | PS 1641 | 2.55^1-9^ | MR | 149 | JSM 188 | 34.69^S-o^ | S | 227 | EC 396065 | 87.70 ^zTzU^ | HS |
| 72 | RKS 47 | 2.55^1-9^ | MR | 150 | AMS 56 | 34.79^R-o^ | S | 228 | CAT 418 | 89.61 ^zU^ | HS |
| 73 | RKS 63 | 2.55^1-9^ | MR | 151 | JS 20-61 | 36.41^T-r^ | S | 229 | EAGLE 81 | 89.61 ^zU^ | HS |
| 74 | RVS 2011-1 | 2.55^1-9^ | MR | 152 | SL96 | 36.53^U-q^ | S | 230 | EC 232019 | 89.61 ^zU^ | HS |
| 75 | RVS 2012-15 | 2.55^1-9^ | MR | 153 | NRC 2755 | 37.02^W-p^ | S | - | - | - | - |
| 76 | SQL 32 | 2.55^1-9^ | MR | 154 | MACS 1370 | 37.79 ^W-s^ | S | - | - | - | - |
| 77 | AGS 76 | 6.12^1-A^ | MR | 155 | EC 572160 | 39.11 ^a-s^ | S | - | - | - | - |
| 78 | AMS 2014-1 | 6.12^1-A^ | MR | 156 | AGS 112 | 39.16 ^a-u^ | S | - | - | - | - |

*Least Significant Difference (LSD); Adjusted means followed by a similar uppercase letter are not significantly different (at p < 0.05)*

**Table S2: Percent Disease Incidence of soybean genotypes during preliminary evaluation in the year 2019. PDI-Percent Disease Incidence, DR-Disease Reaction, HR-Highly Resistant, R- Resistant, MR-Moderately Resistant, MS-Moderately Susceptible, S-Susceptible and HS-Highly Susceptible.**

| S.No | Genotype | PDI | DR | S.No | Genotype | PDI | DR | S.No | Genotype | PDI | DR |
| --- | --- | --- | --- | --- | --- | --- | --- | --- | --- | --- | --- |
| 1 | Harder | 0.000^1^ | HR | 53 | AMS 358 | 0.884^12^ | R | 105 | SQL 32 | 21.678^5-L^ | MS |
| 2 | JS 20-103 | 0.000^1^ | HR | 54 | AMS MB 5-18 | 0.884^12^ | R | 106 | G -10525 | 21.798^6-L^ | MS |
| 3 | JS 20-15 | 0.000^1^ | HR | 55 | CAT 1957 | 0.884^12^ | R | 107 | RVS 2007-06 | 22.635^6-L^ | MS |
| 4 | JS 20-18 | 0.000^1^ | HR | 56 | CAT 2126B | 0.884^12^ | R | 108 | AMS 100-39 | 22.650^6-K^ | MS |
| 5 | JS 20-19 | 0.000^1^ | HR | 57 | CAT 2130 B | 0.884^12^ | R | 109 | CAT 1368 | 22.650^6-K^ | MS |
| 6 | JS 20-20 | 0.000^1^ | HR | 58 | JS 21-06 | 1.194^123^ | MR | 110 | CAT 2503 | 22.819^6-L^ | MS |
| 7 | JS 20-27 | 0.000^1^ | HR | 59 | JS 21-17 | 1.194^123^ | MR | 111 | EC 396055 | 22.819^6-L^ | MS |
| 8 | JS 20-30 | 0.000^1^ | HR | 60 | JS 21-71 | 1.194^123^ | MR | 112 | JSM 298 | 22.891^6-L^ | MS |
| 9 | JS 20-39 | 0.000^1^ | HR | 61 | JS 21-72 | 1.194^123^ | MR | 113 | JS 21-13 | 22.960^6-L^ | MS |
| 10 | JS 20-40 | 0.000^1^ | HR | 62 | JS 21-73 | 1.194^123^ | MR | 114 | EC 389748 | 23.805^7-L^ | MS |
| 11 | JS 20-53 | 0.000^1^ | HR | 63 | JS 21-74 | 1.194^123^ | MR | 115 | PK 768 | 24.471^7-M^ | MS |
| 12 | JS 20-56 | 0.000^1^ | HR | 64 | JS 21-75 | 1.194^123^ | MR | 116 | SL 1074 | 24.471^7-M^ | MS |
| 13 | JS 20-57 | 0.000^1^ | HR | 65 | JS 22-01 | 1.194^123^ | MR | 117 | B 1667 | 24.657^8-N^ | MS |
| 14 | JS 20-71 | 0.000^1^ | HR | 66 | JS 22-02 | 1.194^123^ | MR | 118 | JSM 250 | 24.973^8-O^ | MS |
| 15 | JS 20-73 | 0.000^1^ | HR | 67 | JS 22-04 | 1.194^123^ | MR | 119 | KDS 1073 | 24.973^8-O^ | MS |
| 16 | JS 20-84 | 0.000^1^ | HR | 68 | JS 22-08 | 1.194^123^ | MR | 120 | NRC SL 2 | 24.973^8-O^ | MS |
| 17 | JS 20-90 | 0.000^1^ | HR | 69 | JS 22-09 | 1.194^123^ | MR | 121 | RVS 2011-1 | 25.356^0-P^ | S |
| 18 | JS 20-92 | 0.000^1^ | HR | 70 | JS 99-89 | 1.194^123^ | MR | 122 | MACS 1566 | 25.959^9-Q^ | S |
| 19 | JS 20-96 | 0.000^1^ | HR | 71 | JSM 122 | 1.194^123^ | MR | 123 | B 1664 | 26.543 ^A-R^ | S |
| 20 | JS 20-98 | 0.000^1^ | HR | 72 | JSM 228 | 2.186^1-4^ | MR | 124 | EC 391181 | 26.597 ^B-Q^ | S |
| 21 | JS 21-05 | 0.000^1^ | HR | 73 | JSM 230 | 2.186^1-4^ | MR | 125 | CAT 1847 | 27.449 ^C-R^ | S |
| 22 | JS 21-77 | 0.000^1^ | HR | 74 | JSM 283 | 2.186^1-4^ | MR | 126 | EC 393228 | 27.482 ^C-Q^ | S |
| 23 | MACS 1370 | 0.000^1^ | HR | 75 | JSM 301 | 2.186^1-4^ | MR | 127 | NRC 121 | 27.845 ^D-R^ | S |
| 24 | PI 210178 | 0.000^1^ | HR | 76 | KDS 1097 | 2.186^1-4^ | MR | 128 | JSM 265 | 29.637 ^E-S^ | S |
| 25 | PS 1469 | 0.000^1^ | HR | 77 | PI 204336 | 2.186^1-4^ | MR | 129 | VLS 69 | 30.330 ^F-T^ | S |
| 26 | PS 1611 | 0.000^1^ | HR | 78 | TYPE 49 | 2.186^1-4^ | MR | 130 | JS 335 | 30.374 ^K-R^ | S |
| 27 | PS 1613 | 0.000^1^ | HR | 79 | EC 241690 | 9.130^1-5^ | MR | 131 | NRC 138 | 31.352^G-T^ | S |
| 28 | PS 1641 | 0.000^1^ | HR | 80 | JS 21 -70 | 10.719^2-6^ | MS | 132 | RKS 63 | 31.893 ^H-T^ | S |
| 29 | RSC 10-52 | 0.000^1^ | HR | 81 | EC 34117 | 12.050^2-7^ | MS | 133 | CAT 2310 | 34.095 ^L-U^ | S |
| 30 | SKF SPC 11 | 0.000^1^ | HR | 82 | JS 21-76 | 12.360^2-8^ | MS | 134 | RKS 47 | 37.137 ^N-U^ | S |
| 31 | SL 955 | 0.000^1^ | HR | 83 | JS 20-36 | 13.692^3-0^ | MS | 135 | AMS 38-24 | 37.156 ^M-U^ | S |
| 32 | SL 958 | 0.000^1^ | HR | 84 | RVS 2002-4 | 13.800^3-A^ | MS | 136 | AMS 475 | 37.903 ^P-U^ | S |
| 33 | SL96 | 0.000^1^ | HR | 85 | JS 22-05 | 14.115^4-B^ | MS | 137 | EC 393222 | 39.264 ^R-W^ | S |
| 34 | SQL 89 | 0.000^1^ | HR | 86 | MACS 1520 | 15.107^5-C^ | MS | 138 | AGS-59 | 41.571 ^S-Y^ | S |
| 35 | VLS 94 | 0.000^1^ | HR | 87 | CAT 492 | 15.111^5-D^ | MS | 139 | NRC 99 | 42.147 ^T-Y^ | S |
| 36 | WT 88 | 0.000^1^ | HR | 88 | RSC 11-03 | 15.111^5-D^ | MS | 140 | JS 20-29 | 42.598^U-X^ | S |
| 37 | CAT 2511 | 0.032^12^ | R | 89 | RVS 2009-9 | 15.111^5-D^ | MS | 141 | JS 93-05 | 47.540^W-Z^ | S |
| 38 | CAT 489 A | 0.032^12^ | R | 90 | JS 22-03 | 15.671^5-D^ | MS | 142 | EC 590225 | 50.801 ^X-a^ | HS |
| 39 | CAT 87 | 0.032^12^ | R | 91 | NRC 2755 | 16.341^5-D^ | MS | 143 | JSM 229 | 51.500 ^V-b^ | HS |
| 40 | DS 1318 | 0.032^12^ | R | 92 | NRC 86 | 16.664^5-D^ | MS | 144 | EC 547464 | 56.821 ^Z-c^ | HS |
| 41 | DS 3104 | 0.032^12^ | R | 93 | EC393224 | 17.238^5-E^ | MS | 145 | AGS174 | 56.896 ^Z-d^ | HS |
| 42 | DS 3105 | 0.032^12^ | R | 94 | JS 21-18 | 18.400^5-F^ | MS | 146 | EC 241709 | 62.582 ^b-e^ | HS |
| 43 | DS 3106 | 0.032^12^ | R | 95 | JS 22-06 | 18.400^5-E^ | MS | 147 | NRC 100 | 63.020 ^a-e^ | HS |
| 44 | DSB 25 | 0.032^12^ | R | 96 | JS 97-52 | 18.610^5-G^ | MS | 148 | EC 109543 | 66.259 ^c-e^ | HS |
| 45 | EC 350664 | 0.032^12^ | R | 97 | MACS 1543 | 19.392^5-H^ | MS | 149 | JS 99-78 | 67.421 ^c-f^ | HS |
| 46 | G 305 | 0.884^12^ | R | 98 | NRC 128 | 19.392^5-H^ | MS | 150 | JSM 146 | 67.421 ^c-f^ | HS |
| 47 | AGS-205 | 0.884^12^ | R | 99 | JS 20-23 | 19.563^5-J^ | MS | 151 | JS 95-60 | 69.252 ^e^ | HS |
| 48 | AGS 31 | 0.884^12^ | R | 100 | JS 22-07 | 19.629^5-J^ | MS | 152 | EC 250608 | 69.327 ^d-f^ | HS |
| 49 | AGS 48 | 0.884^12^ | R | 101 | RVS 2012-15 | 19.671^5-I^ | MS | 153 | JS 20-78 | 70.592 ^e-g^ | HS |
| 50 | AGS 76 | 0.884^12^ | R | 102 | AMS 2014-1 | 20.482^5-J^ | MS | 154 | EC 16213 | 78.866 ^f-g^ | HS |
| 51 | AMS 264 | 0.884^12^ | R | 103 | KDS 1009 | 20.621^5-J^ | MS | 155 | JSM 149 | 82.097 ^g^ | HS |
| 52 | AMS 269 | 0.884^12^ | R | 104 | NRC 133 | 20.621^5-J^ | MS | - | - | - | - |

*Least Significant Difference (LSD); Adjusted means followed by a similar uppercase letter are not significantly different (at p < 0.05)*

**Table S3: Year-wise ANOVA of PDI in preliminary evaluation of soybean genotypes**

| **2018** | | | | |
| --- | --- | --- | --- | --- |
| Sources of Variation | DF | MSS | F value | P value |
| Block | 6 | 4447.32^***^ | 150.60 | 2.19e^-14^ |
| Treatment | 229 | 793.28^***^ | 26.86 | 1.43e^-10^ |
| Treatment: Check | 3 | 2565.45^***^ | 86.87 | 6.70e^-11^ |
| Treatment: Test and Test vs. Check | 226 | 769.76^***^ | 26.02 | 1.87e^-10^ |
| Residuals | 18 | 29.52 |  |  |
| **2019** | | | | |
| Sources of Variation | DF | MSS | F value | P value |
| Block | 5 | 897.63^***^ | 62.56 | 1.64e^-9^ |
| Treatment | 154 | 519.79^***^ | 36.22 | 5.88e^-10^ |
| Treatment: Check | 3 | 1580.96^***^ | 110.18 | 1.92e^-10^ |
| Treatment: Test and Test vs. Check | 151 | 498.71^***^ | 34.75 | 8.00e^-10^ |
| Residuals | 15 | 14.34 |  |  |

Significance at *p*<0.001

**Table S4: Year-wise ANOVA of PDI, AUDPC and Grain yield in selected soybean genotypes evaluated. NS- Non- Significant**

| **2020** | | | | | | | | | |
| --- | --- | --- | --- | --- | --- | --- | --- | --- | --- |
| Sources of Variation | DF | PDI | | AUDPC | | | Grain Yield | | |
|  |  | MSS | F  Value | MSS | | F  Value | MSS | | F  Value |
| Genotype | 47 | 1033.00^***^ | 98.40 | 860071.00^***^ | | 450.00 | 53435.00^***^ | | 174.00 |
| Replication | 2 | 19.30^NS^ | 1.84 | 23253.00^NS^ | | 1.70 | 184.00^NS^ | | 0.59 |
| Error | 94 | 10.50 | - | 1911.00 | | - | 307.00 | | - |
| **2021** | | | | | | | | | |
| Sources of Variation | DF | PDI | | AUDPC | | | Grain Yield | | |
|  |  | MSS | F  Value | MSS | F  Value | | MSS | F  Value | |
| Genotype | 47 | 793.00^***^ | 98.40 | 583853.00^***^ | 490.00 | | 61099.00^***^ | 260.00 | |
| Replication | 2 | 22.80^NS^ | 2.61 | 21130.00^NS^ | 0.948 | | 352.00^NS^ | 1.39 | |
| Error | 94 | 8.73 | - | 1192.00 | - | | 235.00 | - | |

^***^Significant at *p*<0.001

**Table S5: Variance components and genetic parameters of the traits: PDI, AUDPC and Grain yield in the selected genotypes evaluated in the years 2020 and 2021.** PDI – Percent Disease Incidence, AUDPC – Area Under Disease Progress Curve, σ^2^_g_ – Genotypic variance, σ^2^_e_ – Residual variance, σ^2^_p_ – Phenotypic variance, H^2^ – Broad – sense heritability, CV_g_ – Genotypic coefficient of variation, CV_r_  – Residual coefficient of variation and CV_g_  / CV_r_ – Ratio between genotypic and residual coefficient of variation.

| **Parameters** | PDI | | AUDPC | | Grain Yield | |
| --- | --- | --- | --- | --- | --- | --- |
|  | 2020 | 2021 | 2020 | 2021 | 2020 | 2021 |
| σ^2^_g_ | 341 | 261 | 286054 | 194220 | 17709 | 20288 |
| σ^2^_e_ | 10.5 | 8.73 | 1911 | 1192 | 307 | 235 |
| σ^2^_p_ | 351 | 270 | 287964 | 195412 | 18016 | 20523 |
| H^2^ (%) | 97.00  (High)^*^ | 96.80  (High) ^*^ | 99.00  (High) ^*^ | 99.00  (High) ^*^ | 98.00  (High) ^*^ | 99.00  (High) ^*^ |
| CV_g_ | 61.10  (High)^#^ | 62.60  (High) ^#^ | 123  (High) ^#^ | 135  (High) ^#^ | 45.20  (High) ^#^ | 38.20  (High) ^#^ |
| CV_r_ | 10.70  (Medium) ^#^ | 11.4  (Medium) ^#^ | 10  (Medium) ^#^ | 10.60 (Medium) ^#^ | 5.95  (Low) ^#^ | 4.11  (Low) ^#^ |
| CV_g_  / CV_r_ | 5.70 | 5.47 | 12.20 | 12.80 | 7.60 | 9.30 |

^*^Categorization has been done as per Robinson (1966); ^#^ Categorization has been done as per Lush (1940)

**Table S6: Genotypic BLUP values for the traits under study during 2020 and 2021**

| S.No | Genotype | Year 2020 | | | Year 2021 | | |
| --- | --- | --- | --- | --- | --- | --- | --- |
|  |  | AUDPC | PDI | Yield | AUDPC | PDI | Yield |
| 1 | Dsb 21 | 2096.3 | 78.9 | 16.6 | 1806.3 | 65.9 | 28.0 |
| 2 | NRC 86 | 300.3 | 33.7 | 338.1 | 219.0 | 25.3 | 438.1 |
| 3 | JS 97-52 | 556.0 | 40.1 | 346.4 | 350.0 | 31.5 | 408.2 |
| 4 | AMS 264 | 97.6 | 16.3 | 394.4 | 38.1 | 13.0 | 511.1 |
| 5 | NRC 128 | 952.0 | 49.9 | 157.5 | 443.5 | 38.1 | 321.9 |
| 6 | JS 20-96 | 38.4 | 11.8 | 460.7 | 22.5 | 7.0 | 443.1 |
| 7 | PS 1225 | 110.1 | 22.5 | 382.8 | 78.6 | 15.5 | 446.4 |
| 8 | NRC 2755 | 1011.2 | 50.6 | 101.1 | 711.7 | 43.8 | 209.0 |
| 9 | SL 955 | 107.0 | 22.1 | 386.1 | 106.7 | 18.5 | 486.2 |
| 10 | JS 335 | 231.7 | 33.7 | 270.1 | 403.0 | 31.8 | 414.8 |
| 11 | JS 95-60 | 2355.1 | 81.1 | 19.9 | 2133.8 | 75.0 | 18.0 |
| 12 | JS 20-19 | 44.6 | 9.1 | 319.9 | 50.6 | 11.9 | 365.0 |
| 13 | PS 1641 | 44.6 | 14.0 | 422.6 | 103.6 | 18.1 | 366.7 |
| 14 | CAT 87 | 568.5 | 35.2 | 227.1 | 212.7 | 27.1 | 371.7 |
| 15 | AMS MB-5-18 | 350.2 | 33.2 | 351.3 | 658.7 | 40.8 | 285.3 |
| 16 | JS 20-73 | 41.5 | 13.5 | 391.1 | 38.1 | 12.6 | 371.7 |
| 17 | JS 21-71 | 54.0 | 13.3 | 389.5 | 47.4 | 12.3 | 476.3 |
| 18 | CAT 492 | 846.0 | 46.5 | 126.0 | 431.0 | 37.1 | 232.2 |
| 19 | JS 21-05 | 19.7 | 6.3 | 513.7 | 10.0 | 2.4 | 597.5 |
| 20 | JS 20-53 | 54.0 | 11.3 | 331.5 | 44.3 | 8.8 | 458.0 |
| 21 | JS 20-20 | 10.3 | 2.4 | 387.8 | 0.7 | 0.3 | 431.4 |
| 22 | CAT 1957 | 1008.1 | 48.4 | 150.8 | 365.5 | 32.9 | 228.9 |
| 23 | JS 20-30 | 730.6 | 39.6 | 160.8 | 215.9 | 28.5 | 260.4 |
| 24 | JS 21-77 | 428.1 | 33.9 | 379.5 | 381.1 | 30.4 | 353.4 |
| 25 | MACS 1370 | 412.6 | 38.6 | 175.7 | 449.7 | 40.0 | 287.0 |
| 26 | HARDER | 378.3 | 41.8 | 179.0 | 166.0 | 28.0 | 371.7 |
| 27 | JS 21-73 | 82.0 | 16.8 | 450.8 | 125.4 | 20.0 | 532.7 |
| 28 | JS 20-98 | 22.8 | 8.3 | 510.4 | 16.3 | 7.0 | 630.7 |
| 29 | JS 20-29 | 1503.9 | 68.4 | 43.1 | 880.1 | 52.0 | 130.9 |
| 30 | MACS 1520 | 275.4 | 29.4 | 308.3 | 409.2 | 36.9 | 355.1 |
| 31 | EC 393228 | 450.0 | 37.2 | 192.3 | 717.9 | 49.1 | 162.5 |
| 32 | KDS 1097 | 406.3 | 32.1 | 313.2 | 141.0 | 19.6 | 519.4 |
| 33 | JS 20-39 | 54.0 | 12.5 | 382.8 | 84.9 | 14.2 | 453.0 |
| 34 | JS 93-05 | 1566.2 | 59.7 | 29.9 | 1360.4 | 52.5 | 81.1 |
| 35 | PS 1613 | 658.9 | 38.9 | 286.7 | 256.4 | 28.8 | 466.3 |
| 36 | EC 350664 | 462.4 | 33.7 | 177.3 | 240.8 | 25.6 | 265.4 |
| 37 | DS 1318 | 44.6 | 13.0 | 351.3 | 41.2 | 13.5 | 497.9 |
| 38 | EC 34117 | 150.6 | 22.1 | 291.7 | 247.0 | 28.0 | 270.4 |
| 39 | PK 768 | 57.1 | 15.0 | 402.7 | 66.2 | 14.6 | 461.3 |
| 40 | AMS 100-39 | 440.6 | 35.4 | 296.7 | 222.1 | 25.0 | 418.2 |
| 41 | JSM 283 | 347.1 | 31.3 | 232.0 | 509.0 | 36.4 | 265.4 |
| 42 | JSM 228 | 44.6 | 14.0 | 361.3 | 59.9 | 11.2 | 408.2 |
| 43 | JS 22-01 | 19.7 | 6.3 | 500.5 | 6.9 | 3.3 | 579.2 |
| 44 | NRC 138 | 814.8 | 49.4 | 132.6 | 487.2 | 33.6 | 321.9 |
| 45 | DS 3106 | 163.1 | 24.1 | 411.0 | 41.2 | 13.3 | 534.4 |
| 46 | DS 3104 | 184.9 | 25.1 | 321.5 | 119.2 | 21.3 | 453.0 |
| 47 | CAT 1847 | 178.7 | 26.2 | 339.7 | 131.6 | 24.7 | 365.0 |
| 48 | PS 1611 | 119.5 | 23.4 | 402.7 | 35.0 | 12.2 | 547.7 |
